# Oral Antioxidant Vitamins and Magnesium Limit Noise-Induced Hearing Loss by Promoting Sensory Hair Cell Survival: Role of Antioxidant Enzymes and Apoptosis Genes

**DOI:** 10.3390/antiox9121177

**Published:** 2020-11-25

**Authors:** Juan C. Alvarado, Verónica Fuentes-Santamaría, Pedro Melgar-Rojas, María C. Gabaldón-Ull, José J. Cabanes-Sanchis, José M. Juiz

**Affiliations:** 1 Instituto de Investigación en Discapacidades Neurológicas (IDINE), School of Medicine, Universidad de Castilla-La Mancha, 02008 Albacete, Spain; veronica.fuentes@uclm.es (V.F.-S.); pmelgarrojas@gmail.com (P.M.-R.); mcruz.gabaldon@uclm.es (M.C.G.-U.); josejulio.cabanes@uclm.es (J.J.C.-S.); 2 Department of Otolaryngology, Hannover Medical School, NIFE-VIANNA, Cluster of Excellence Hearing4all-German Research Foundation, 30625 Hannover, Germany

**Keywords:** auditory, deafness, acoustic trauma, hair cells, antioxidant, otoprotection

## Abstract

Noise induces oxidative stress in the cochlea followed by sensory cell death and hearing loss. The proof of principle that injections of antioxidant vitamins and Mg^2+^ prevent noise-induced hearing loss (NIHL) has been established. However, effectiveness of oral administration remains controversial and otoprotection mechanisms are unclear. Using auditory evoked potentials, quantitative PCR, and immunocytochemistry, we explored effects of oral administration of vitamins A, C, E, and Mg^2+^ (ACEMg) on auditory function and sensory cell survival following NIHL in rats. Oral ACEMg reduced auditory thresholds shifts after NIHL. Improved auditory function correlated with increased survival of sensory outer hair cells. In parallel, oral ACEMg modulated the expression timeline of antioxidant enzymes in the cochlea after NIHL. There was increased expression of glutathione peroxidase-1 and catalase at 1 and 10 days, respectively. Also, pro-apoptotic caspase-3 and Bax levels were diminished in ACEMg-treated rats, at 10 and 30 days, respectively, following noise overstimulation, whereas, at day 10 after noise exposure, the levels of anti-apoptotic Bcl-2, were significantly increased. Therefore, oral ACEMg improves auditory function by limiting sensory hair cell death in the auditory receptor following NIHL. Regulation of the expression of antioxidant enzymes and apoptosis-related proteins in cochlear structures is involved in such an otoprotective mechanism.

## 1. Introduction

Excess mechanical energy carried by loud sounds damages the auditory sensory neuroepithelium and associated peripheral and central neurons and their connections, leading to hearing loss [1,2]. Acoustic trauma or noise-induced hearing loss (NIHL) is the result of exposure to environmental noise, potentiated by complex genetic susceptibility [2]. According to data from the National Health and Nutrition Examination Survey (NHANES), 24% of adults in the U.S.A., aged 20 to 69, have audiometric findings compatible with NIHL [3]. It is estimated that more than 600 million people worldwide are at risk of hearing loss from exposure to both occupational and recreational noise sources, which makes NIHL a major health problem [2]. Hence the need for therapeutic approaches that, along with physical barrier, noise-exposure prevention measures, may contribute to effectively prevent or treat NIHL.

An important leap forward in the understanding of NIHL has been the realization that damage to inner ear structures has metabolic and biochemical foundations [1,2]. Whereas direct mechanical fracture of auditory structures is relevant after impulse noise, damage induced by continuous noise, which is more frequent, involves several interconnected pathophysiological changes [4]. Acoustic overstimulation of auditory hair cells causes abnormally large ion fluxes through ion channels in the cell membrane, notably excess damaging (see below) Ca^2+^ inflow. Overstimulated hair cells release large amounts of glutamate neurotransmitter, which may contribute to damage by causing excitotoxicity on primary sensory neurons. Noise also alters cochlear blood flow and induces inflammation and oxidative stress in a self-perpetuating cycle [4,5]. Actually, such converging mechanisms further potentiate oxidative stress, with excess accumulation of highly reactive toxic free radicals, which in turn leads to the activation of a cascade of signaling pathways leading to cell death. Sensory hair cell death, caused by apoptosis, necrosis, or other mechanisms [6] is not followed naturally by regenerative replacement in mammals, which eventually results in NIHL becoming irreversible.

More specifically, a currently accepted mechanistic sequence is that noise overstimulation increases the displacement rate of mechano-sensory stereocilia in hair cells, which initiates large ion fluxes through mechanosensitive channels across the membrane [7]. This increases mitochondrial demands of ATP synthesis to sustain ion homeostasis. Increased electron transfer through the electron transport chain causes, in turn, ‘leakage’ of electrons (e^−^). One consequence is that O_2,_ the final e^−^ acceptor in the redox chain coupled to ATP synthesis, is incompletely reduced by one e^−^. This leads to the generation of large amounts of the superoxide anion (O_2_^•−^), a free radical in the form of a reactive oxygen species (ROS). O_2_^•−^ is the precursor of other ROS, like H_2_O_2_ and other peroxides, the hydroxyl radical (OH) or singlet oxygen [8]. Further combinations with nitrogen derivatives render reactive nitrogen species (RNS), notably peroxynitrites. ROS/RNS have large oxidative potential due to its free e^−^. Excess ROS/RNS override antioxidant defenses, leading to oxidative stress due to toxicity derived from multiplicative redox processes. ROS/RNS oxidize amino acids in proteins, so that many key enzymes are inactivated. Importantly, they also cause lipid peroxidation, with structural damage to membranes, and produce oxidative damage to DNA and RNA [8]. Cells in the auditory receptor, including sensory hair cells, seem particularly prone to extreme oxidative stress. This is so because it is conceivable that antioxidation defensive mechanisms are working close to limits even under normal conditions, due to the intrinsically high metabolic energy demands of the auditory transduction mechanism, which generates ROS as a by-product, and also because mitochondrial Ca^2+^ overload may lead directly by itself to excess ROS generation through enzymatic dysregulations [6,9].

Oxidative stress is regulated and limited by interrelated enzymatic and non-enzymatic mechanisms, normally restoring ROS/RNS to physiological levels [10]. Among enzymatic mechanisms, superoxide dismutase (SOD1, 2, and 3, in the cytosol, mitochondria, and extracellular space respectively) catalyze dismutation of O_2_^•ˉ^ to H_2_O_2_. Catalase (CAT), in turn, catalyzes fast conversion of H_2_O_2_ in H_2_O and O_2_. Glutathione peroxidase (GPX1, 8 isoforms), on the other hand, inactivates peroxides, including H_2_O_2_ and lipid peroxides, thus protecting membranes from oxidation [10,11]. Among non-enzymatic antioxidants, besides the central role of glutathione, antioxidant vitamins, notably vitamins C, E, and carotenoids are of great relevance. Vitamin E reduces peroxyl radicals in lipid bilayers. Vitamin C eliminates free radicals in aqueous phase and assists in regenerating oxidized vitamin E. Beta-carotenoids also prevent lipid peroxidation and remove highly reactive singlet oxygen [11].

The mechanistic understanding outlined above has fostered considerable interest in the use of antioxidants against noise damage to the auditory receptor. Several antioxidant substances have been tried under different approaches, mostly at the stage of proof of concept, reinforcing the notion of a causal effect of oxidative stress in NIHL. Thus, exogenous regulation and regeneration of glutathione levels [12,13]; potentiation of antioxidant enzymes such as GPX by using enzyme mimics such as ebselen [14,15,16]; or free-radical scavengers such as sulfur-containing amino acids and derivatives including d-methionine, or N-acetyl-cysteine, which are direct antioxidants besides its role in glutathione regeneration, have shown otoprotective potential in NIHL [17,18]. Among free-radical scavengers, antioxidant vitamins, in particular vitamins C and E, also have been shown to protect the auditory receptor from NIHL damage [19,20,21].

A powerful otoprotective potential of a combination of antioxidant vitamins (A, C, and E) along with Mg^2+^ has been proposed [20,22]. It has been suggested that the different antioxidation mechanisms of each vitamin combined with cochlear vasodilation induced by Mg^2+^, along with other effects of this cation mainly related to Ca^2+^ antagonism, give rise to a synergistic interaction resulting in efficient otoprotection against NIHL [20]. However, otoprotective mechanisms of orally administered ACEMg have not been investigated, and it is even unclear whether increased survival of sensory hair cells may be involved [20,22]. This should give new insights about antioxidant otoprotection in NIHL using administration routes closer to clinical applications. Towards this end, we have tested the otoprotective role of an oral combination of vitamins A, C, E, and Mg^2+^ (ACEMg) against permanent NIHL in relation to auditory sensory cell survival and cellular changes in the expression and distribution of molecules involved in antioxidation and apoptotic mechanisms in the cochlea

## 2. Materials and Methods

### 2.1. Experimental Animals

Young adult (three-month-old) Wistar rats (*n* = 48) from Charles River Laboratories (Barcelona, Spain), maintained on a 12 h light/dark cycle with food and water ad libitum at the Universidad of Castilla-La Mancha Animal House facility (Albacete, Spain) were used. Sample size was calculated using the LaMorte’s power calculation spreadsheet from Boston University [23]. The procedures involving the use and care of the animals were approved by the corresponding Institutional Animal Care and Use Committee (permit no. PR-2013-02-03). These protocols were in accordance with the guidelines of the European Communities Council (Directive 2010/63/EU) and current national legislation (R.D. 53/2013; Law 32/2007).

### 2.2. Antioxidant (ACEMg) Supplementation

Rats were initially divided into two groups, one fed with regular chow (“normal diet,” ND, *n* *=* 24), and the other with chow enriched with a combination of vitamin A, vitamin C, vitamin E, and Mg^2+^ (“enriched diet,” ED, *n* *=* 24) (Harlan Teklad Diet TD.110032) [20,22]. The ED consisted in a tocopherol-stripped soy-based diet supplemented with b-carotene (vitamin A precursor, 1.05 g/kg), vitamin C (10.29 g/kg), vitamin E (7.76 g/kg), and MgSO_4_ (Mg, 13.48 g/kg). Feeding with ED began 10 days before noise overexposure (see next section) and was maintained until the end of the experiments. The amount of chow was weighted daily to control an equivalent range of chow intake across groups throughout the duration of the experiments.

### 2.3. Noise Exposure Protocol

The noise stimulation protocol consisted of exposure to broadband noise (118 dB sound pressure level, SPL), for 4 h a day over 4 consecutive days. The sound was delivered inside a methacrylate reverberating chamber of 60 (length) × 70 (width) × 40 cm (height) with tilted and non-parallel walls to ensure a more homogeneous sound field and to limit standing waves [24]. The chamber was placed into a double-walled sound-attenuating booth located inside a sound-attenuating room. During noise exposure animals were awake, could move freely in the chamber and had free access to water. Noise-exposed animals, fed either with ND or ED (see preceding section), were randomly assigned to survival groups of 1 day (ND-1D, *n* = 6 and ED-1D, *n* = 6), 10 days (ND-10D, *n* = 6 and ED-10D, *n* = 6), and 30 days (ND-30D, *n* = 6 and ED-30D, *n* = 6) post-noise exposure. At the end of the corresponding survival times, animals underwent either qPCR or cochlear histology and immunocytochemistry, as described further in detail. Age-matched rats not exposed to noise, fed with ND or ED were used as controls (ND-CTR, *n* = 6 and ED-CTR, *n* = 6).

### 2.4. Auditory Brainstem Response (ABR) Recordings

ABRs were recorded from ND and ED-fed, noise-exposed experimental animals, the day before the beginning of the noise exposure and at the end of each survival time point, as well as from noise-unexposed control animals (ND-CTR and ED-CTR, see above). Recordings were conducted as described previously in detail [25,26,27]. Testing took place in a sound-attenuating, electrically shielded booth (EYMASA/INCOTRON S.L., Barcelona, Spain) located inside a sound-attenuating room. To perform the ABR recordings, rats were anesthetized with 4% isoflurane (1 L/min O_2_ flow rate, Esteve Pharmaceuticals, Barcelona, Spain) for induction and 1.5–2% for maintenance. During recordings body temperature was maintained at 37.5 ± 1 °C, using a non-electrical heating pad, and monitored with a rectal probe. The electrodes (subdermal needles from Rochester Electro-Medical, Tampa, FL, USA) were positioned at the vertex (non-inverting) and at the right (inverting) and left (ground) mastoids. Sound stimulation and recordings were performed using a BioSig System III (Tucker-Davis Technologies, Alachua, FL, USA). The acoustic stimuli consisted of pure tone burst sounds (5 ms rise/fall time without a plateau with a cos2 envelope delivered at 20/s) at seven different frequencies (0.5, 1, 2, 4, 8, 16, and 32 kHz). The stimuli, generated digitally by the SigGenRP software (Tucker-Davis Technologies, Alachua, FL, USA) and the RX6 Piranha Multifunction Processor (Tucker-Davis Technologies, Alachua, FL, USA), were delivered into the right ear using an EDC1 electrostatic speaker driver (Tucker-Davis Technologies) through an EC-1 electrostatic speaker (Tucker-Davis Technologies). Calibration was performed prior to the experiments using SigCal software (Tucker-Davis Technologies) and an ER-10B+ low noise microphone system (Etymotic Research Inc., Elk, Groove, IL, USA). All evoked responses were filtered (0.3–3.0 kHz), averaged (500 waveforms) and stored for offline analysis.

#### ABR Data Analysis

Measurement of auditory thresholds was conducted by recording evoked responses from 80 dB SPL in descending 5 dB steps. For each frequency tested, the auditory threshold was defined as the stimulus intensity that evoked waveforms with a peak-to-peak voltage >2 standard deviations from the background activity measured before the stimulus onset [25,28,29]. The maximum intensity level was 80 dB [25,28,30,31] to reduce chances of inducing acoustic trauma in unexposed animals and additional overstimulation in noise-exposed rats during the ABR recordings. Following the noise stimulation protocol, if no evoked responses were obtained at 80 dB, the auditory thresholds were set at that value for statistical purposes [25,28,31,32,33,34]. The threshold shift was defined as the numerical difference between the auditory thresholds following the noise overstimulation, minus the auditory thresholds in the noise-unexposed condition, for each animal at each of the frequencies tested [25,28].

### 2.5. Real Time-Quantitative Polymerase Chain Reaction (qPCR)

#### 2.5.1. Cochlear Dissection and RNA Extraction

Both noise-unexposed, ND (*n* = 3) and ED (*n* = 3) fed rats (ND-CTR and ED-CTR) and noise-exposed rats at the defined time points (ND-1D, *n* = 3 and ED-1D, *n* = 3*;* ND-10D, *n* = 3 and ED-10D, *n* = 3*;* ND-30D, *n* = 3 and ED-30D, *n* = 3) were deeply anesthetized with 1.5–2% isoflurane (1 L/min O_2_ flow rate) (Esteve Pharmaceuticals, Barcelona, Spain) followed by an intraperitoneal injection of a combination of ketamine (80 mg/kg) (Pfizer Inc., New York, NY, USA) and xylazine (10 mg/kg) (Calier, S.A., Barcelona, Spain) After euthanasia, temporal bones were rapidly removed and placed in cold 1× phosphate-buffered saline (PBS). Whole cochleae (including the Organ of Corti, lateral wall tissues and modiolar portion of the VIIIth nerve) were isolated within 8–10 min using a dissection microscope, collected into cryotubes (Corning Inc., Corning, NY, USA) and rapidly frozen on dry ice. Frozen cochleae were weighed and placed on the corresponding volume of cold TRIzol reagent (Thermo Fisher Scientific, Waltham, MA, USA). They were quickly homogenized using a Polytron PT 2100 homogenizer (Dispersing aggregate PT-DA 2105/2EC; Rotor–Ø 3 mm) (Kinematica, Luzern, Switzerland) at 30 × 1000 rpm for <30 s. Total RNA was extracted according to TRIzol reagent manufacturer’s instructions. Quantity and quality of RNAs was assessed by electrophotometry (Nanodrop ND-1000, Thermo Fisher Scientific) and electrophoresis. One randomly chosen cochlea from each animal was used in the next steps. RNAs were stored at −80 °C.

#### 2.5.2. cDNA Synthesis and qPCR

Messenger RNA expression was analyzed by reverse transcription followed by qPCR, as described elsewhere [31]. Briefly, RevertAid First Strand cDNA Synthesis Kit (Thermo Fisher Scientific) was used to synthesize first strand cDNAs from 1 µg of RNA using oligo–(dT)18 as primer, following manufacturer’s instructions. After the reaction, cDNAs were diluted 10-fold. qPCR was performed in a One Step Plus Real-Time PCR System from Applied Biosystems^TM^, (Thermo Fisher Scientific) using Fast SYBR Green Master Mix (Thermo Fisher Scientific) as reagent. The ‘master mix’ (MM) for one well of the qPCR plate contained: 2.8 µL of sterile H_2_O MilliQ, plus 0.1 µL of each primer (previously resuspended at 10 µM), plus 5 µL of Fast SYBR Green Master Mix. 28 µL of the MM and 7 µL of the corresponding cDNA were mixed to make the ‘reaction mix’ (RM) in three wells of each plate used for qPCR. Finally, 10 µL of the RM were dispensed into three wells of each plate to make the reactions. Quantitative PCR was performed using specific primer pairs for amplifying transcripts of interest (Table 1).

Primer pairs were tested to verify specificity for the target gene by BLAST analysis (NCBI) and matched against the genomic sequence, downloaded from Ensembl Data Base (Vega), to check their selectivity for the cDNA sequence. Quantification of expression (expressed as fold change) from the Cq data was calculated using the Step One Software v2.3 from Applied Biosystems^TM^ (Thermo Fisher Scientific) following the ∆∆Cq method [35]. In summary, the expression level of a target gene was first normalized to the average level [36] of the best reference gene pair for cochlear tissues (Hprt1/Tbp; [31]) to obtain the ∆Cq value of each gene in the samples (control and noise-exposed). Then, the ∆∆Cq of each gene was calculated as: ∆Cq (noise-exposed group) – ∆Cq (control group), where ‘noise-exposed group’ corresponds to each experimental group detailed above. Relative expressions expressed as fold changes were calculated according to the equation

Fold change = 2 − ΔΔCq(1)

### 2.6. Cochlear Whole-Mount Preparations

ND and ED noise-exposed (ND-1D, *n* = 3 and ED-1D, *n* = 3*,* ND-10D, *n* = 3 and ED-10D, *n* = 3*;* ND-30D, *n* = 3 and ED-30D, *n* = 3) and unexposed (ND-CTR, *n* = 3 and ED-CTR, *n* = 3) rats were terminally anesthetized with an intraperitoneal injection of sodium pentobarbital, (200 mg/Kg) and perfused intracardially with 0.9% saline wash followed by a 4% paraformaldehyde solution diluted in 0.1 M phosphate buffer (PB, pH 7.3). As previously described [27,31], the left cochleae were removed and decalcified in 50% RDO rapid decalcification solution (Apex Engineering Products Corp., Aurora, IL, USA) for 2 h and the organ of Corti was isolated and dissected into individual turns. Cochlear turns were mounted on glass slides, counterstained with DAPI nuclear staining and cover slipped. Fluorescence was visualized using a laser scanning confocal microscope (LSM 710; Zeiss, Jena, Germany) equipped with a 40X Plan Apo oil-immersion objective (1.4 NA) and excitation laser lines at 405 and 594 nm. Series of Z-stack confocal microscopy images (3–5 μm thickness, 1024 × 1024 pixels) were acquired at intervals of 0.5 μm and saved as TIFF files. Outer hair cell (OHC) counts were performed in segments of approximately 250 μm-long along the length of the organ of Corti, using the public domain image analysis software Scion Image for Windows (version beta 4.0.2; Scion Corp., Frederick, MD, USA.) [37,38]. OHC survival following noise exposure was expressed as the percentage of remaining OHC along the length of the basilar membrane relative to the noise-unexposed control condition [37,38,39].

### 2.7. Cochlear Immunohistochemistry

In both ND and ED noise-exposed and ND-CTR and ED-CTR unexposed rats, the right cochleae were cryoprotected in 30% sucrose in PBS, frozen at −70 °C by immersion in a 2-propanol/dry ice bath, and further encased in a 15% sucrose and 10% gelatin solution. Blocks were sectioned parallel to the modiolus in a cryostat at a thickness of 20 mm. Sections in the modiolar plane were mounted serially on gelatin-coated slides and processed for immunohistochemistry. After several rinses in PBS containing 0.2% Triton X-100 (Tx), sections were incubated for 1 h in PBS-Tx (0.2%) with 10% normal goat serum (NGS). Next, sections were incubated overnight in a humid chamber at 4 °C with the corresponding primary antibodies (anti-CAT, GPX1, SOD1, and BCL-2; see Table 2) diluted in a solution containing PBS-Tx (0.2%), pH 7.4. The next day, after four 15 min rinses in PBS-Tx (0.2%), sections were incubated for 2 h in the corresponding fluorescent secondary antibody conjugated to Alexa 488 (1:200, Vector Laboratories, Burlingame, CA, USA) and also in biotinylated phalloidin (Pha) which was then visualized with streptavidin conjugated to Alexa 594 (Molecular Probes, Eugene, OR, USA). Finally, sections were counterstained with DAPI nuclear staining and cover slipped. Immunofluorescence was visualized using a laser scanning confocal microscope as outlined in the previous section.

### 2.8. Statistical Analysis

Data are expressed as mean ± SEM. Measurements of ABR parameters were performed at 80 dB SPL unless otherwise indicated. Two-way repeated measures analysis of variance (ANOVA) with diet (normal diet, ND vs. enriched diet, ED) as an independent variable and time points (control, 1 day, 10 days and 30 days) as a repeated independent variable was used. For each frequency studied, the possible statistically significant main effect of the diet over the survival time was evaluated. If the main analysis indicated a significant effect of one factor or an interaction between factors, a Scheffé post hoc analysis was performed. Significance levels (α) and power (β) were set to 0.05 and 95%, respectively.

### 2.9. Preparation of Figures

Photoshop (Adobe v5.5) and Canvas (Deneba v6.0) software packages were used to adjust the size, brightness, and contrast of the images used for the figures in this publication.

## 3. Results

### 3.1. ACEMg Otoprotection Against NIHL: ABR Recordings

**Figure 1:**
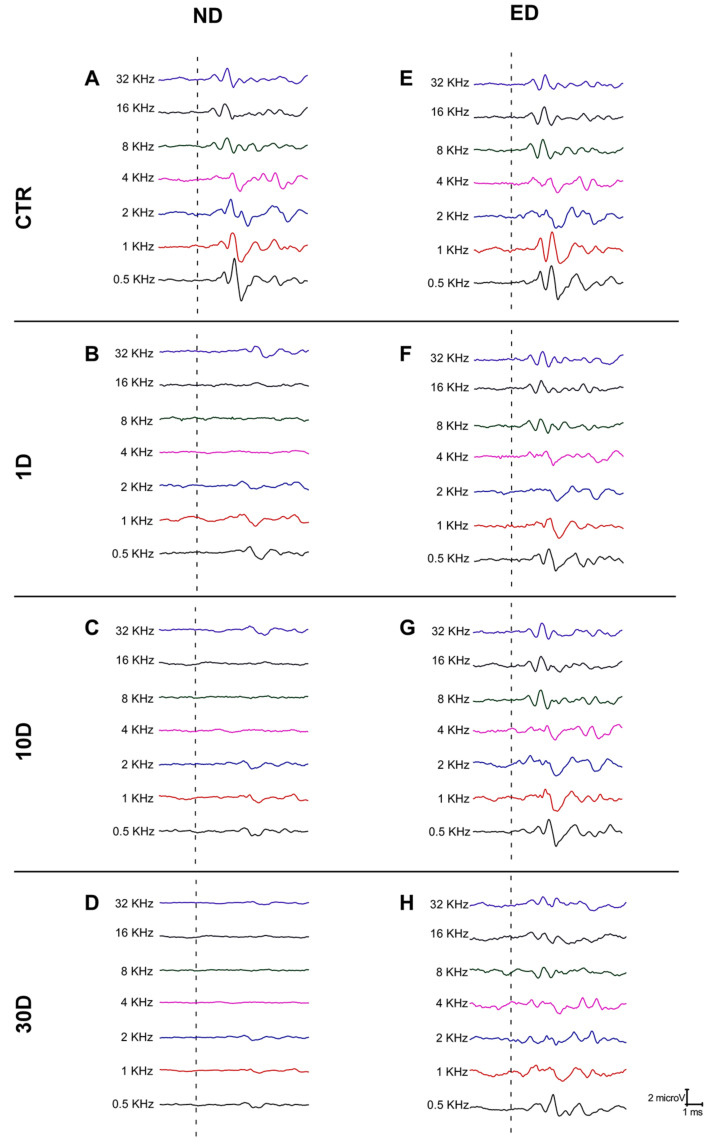
Representative ABR recordings from rats fed with normal diet (ND) and ACEMg-enriched diet (ED) after noise-induced hearing loss. Line graphs illustrating examples of ABR recordings from control animals fed with ‘normal diet’ (ND) (left column) or ‘enriched diet’ (ED)-fed (right column). ND-CTR and ED-CTR are control ABRs from animals not exposed to noise. Both ND-CTR (**A**) and ED-CTR (**E**) rats showed similar recording traces, with the characteristic 4 to 5 evoked waveforms. After the noise exposure protocol, there was an almost complete absence of the typical ABR waveforms at all frequencies in the ND-1D (**B**), ND-10D (**C**), and ND-30D (**D**) survival groups. However, in the ED-fed rats, oral administration of ACEMg preserved the evoked waves at 1D (**F**), 10D (**G**), and 30D (**H**) after the noise overexposure, although there was a reduction in the waveform amplitudes. Dashed lines indicate stimulus onset. Stimulus intensity = 80 dB SPL.

As illustrated in Figure 1, and consistent with previous studies [24,25,34], ABR recordings from both ND-CTR and ED-CTR rats (Figure 1A,E), not exposed to noise, showed the usual pattern of 4 to 5 evoked waveforms following the stimulus onset, where wave II was the largest, followed by waves I, IV, V, and III. Similar to what has been described elsewhere in rats [24,31], ABR recordings carried out at 1 day (Figure 1B), 10 days (Figure 1C), and 30 days (Figure 1D) after noise exposure in the ND rats, showed lack of evoked responses at any time point and frequency evaluated. In contrast, in the ED animals evoked response waves were still present at all frequencies tested and at all time points studied, albeit with reduced amplitudes (Figure 1F–H).

#### 3.1.1. Auditory Thresholds and Threshold Shift after NIHL

**Figure 2:**
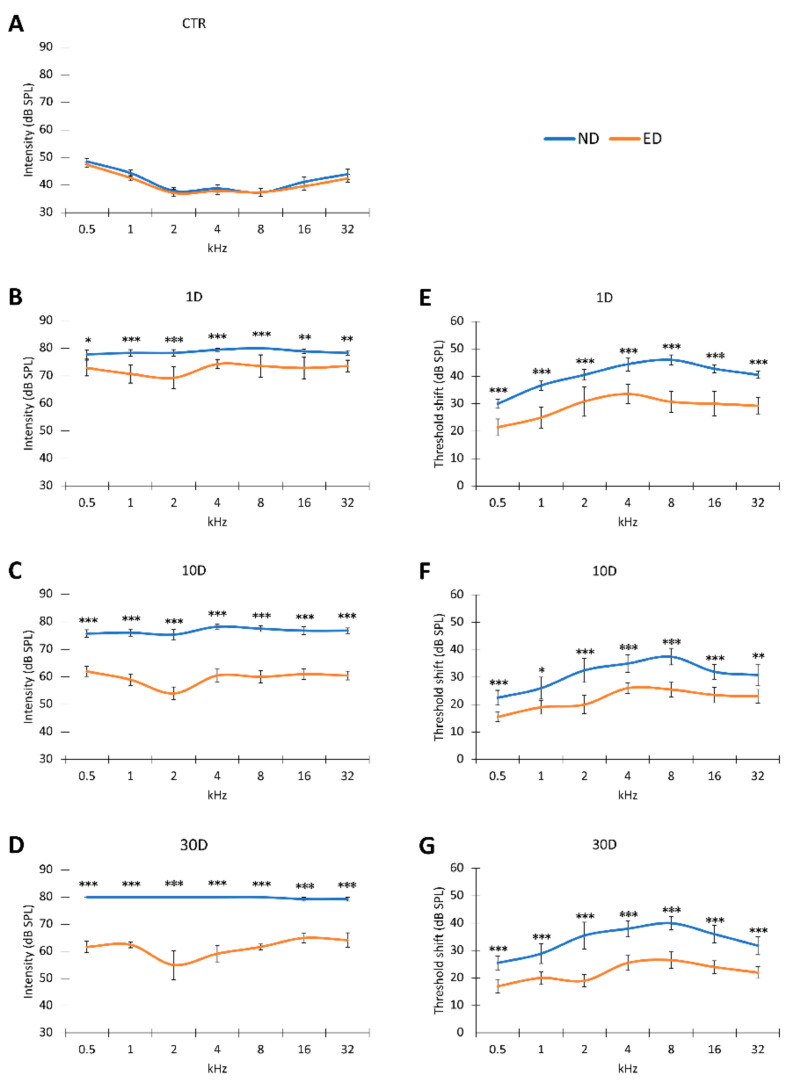
Line graphs showing auditory thresholds and threshold shifts in ND and ED animals after noise-induced hearing loss. Following the noise overexposure protocol, the auditory thresholds in both ND and ED rats (**B**–**D**) were increased at all frequencies compared to the control condition (see both ND-CTR and ED-CTR in (**A**)). However, the mean values in the ED groups were significantly lower than those found in ND rats (**B**–**D**) with substantial recovery at 10D (**C**) and 30D (**D**) after the exposure. This observation was corroborated with the threshold shifts, which were also significantly lower in ED-fed than in ND-fed rats (**E**–**G**). * *p* < 0.05; ** *p* < 0.01; *** *p* < 0.001.

In control animals not exposed to noise (ND-CTR and ED-CTR), the average auditory thresholds were higher at the lowest frequencies, with values of 48 dBs at 0.5 kHz, lower at medium frequencies, with values of 37 dBs at 8 kHz and augmented again at the highest frequencies tested, with values around 45 dBs at 32 kHz (Figure 2A, Table 3) [24,25,34]. No differences were evident between ND-CTR and ED-CTR animals. In noise-exposed ND-fed rats, absolute hearing thresholds at 1 day, 10 days, and 30 days after noise-exposure were, statistically, significantly higher than those in noise-unexposed (ND-CTR or ED-CTR) animals and very similar across frequencies (Figure 2B–D, Table 3). Actually, at all post-noise exposure time points and frequencies evaluated, the average thresholds in noise-exposed, ND-fed rats, were consistently above 75 dB SPL. Threshold shifts in these animals ranged from 22 to 46 dB, and there was no significant recovery of auditory threshold values at any survival time or frequency tested, indicating that noise-exposed rats fed with ND had permanent hearing loss in response to the repeated noise exposure protocol used (Figure 2E–G, Table 3). Similar findings have been reported elsewhere with an identical noise stimulation protocol [24,31].

#### 3.1.2. Recovery of Auditory Thresholds and Threshold Shifts after Oral Administration of ACEMg

In noise-exposed ED-fed rats, there was a preservation of auditory thresholds, which were significantly lower than those seen in noise-exposed ND-fed rats at the same survival times (Figure 2B–D). At 10 (Figure 2C, Table 3) and 30 (Figure 2D, Table 3) days of survival, threshold recovery was significantly larger, compared to the values found at 1 day (Figure 2B, Table 3). In spite of significant recovery, mean threshold values still were significantly higher than those in unexposed controls (ND-CTR or ED-CTR) (compared Figure 2A and Figure 2B–D, Table 3).

Similarly, threshold shifts in the noise-exposed ED-fed animal group also were significantly lower than those in the noise-exposed ND-fed animals. Threshold recoveries in ED-10D (Figure 2F, Table 3) and ED-30D (Figure 2G, Table 3) rats were more evident at 2, 4, and 8 kHz (Figure 2E–G, Table 3). Even though there was a still a permanent threshold shift in the noise-exposed rats fed with ED, oral administration of ACEMg preserves wave morphology in the ABR recordings and considerably reduces threshold shifts following acute noise overexposure.

### 3.2. Loss of OHCs in the Cochlea after NIHL and ACEMg Otoprotection

#### 3.2.1. Noise-Exposed, Untreated Rats

**Figure 3:**
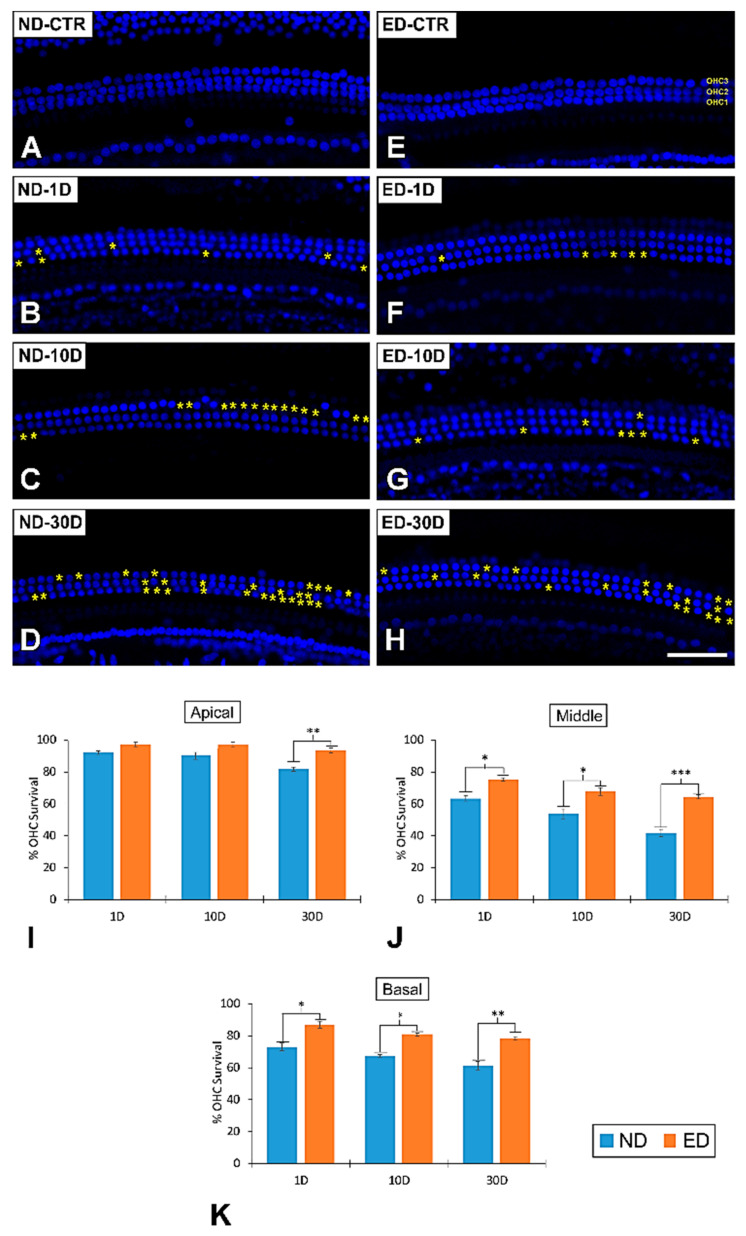
Surface preparation images illustrating DAPI staining in the middle cochlear turn in ND-fed and ED-fed control and noise-exposed animals. There was an increase in hair cells loss at all post-exposure survival times in ND-fed animals (**B**–**D**) compared to the control condition (**A**). Following oral administration of ACEMg (**E**–**H**), there was an increase in hair cell survival in comparison to non-treated animals at the same survival time. Yellow asterisks show loss of OHCs at 1D, 10D, and 30D postexposure. (**I**–**K**) Bar graphs illustrating percentage of OHC loss following noise exposure and oral ACEMg treatment. In the apical cochlear turn (**I**), a significant loss of OHCs was evident only in the ND-30D group when compared to ED-30D rats. After noise exposure, in the middle (**J**) and basal (**K**) cochlear turns, there was a significant reduction in the number of OHCs at 1, 10, and 30 days in both ND and ED rats. However, in the ED rats OHC loss was significantly lower at all survival times when compared to ND animals. Asterisks indicate significant differences between ND and ED rats. ^∗^ *p* < 0.05; ^∗∗^ *p* < 0.01, ^∗∗∗^ *p* < 0.001. Scale bar = 50 µm is shown in H.

Outer hair cell death following noise exposure in the ND-fed rats, relative to noise-unexposed control animals was more prominent in both the middle (Figure 3A–D) and basal turns of the cochlea and occurred earlier than in the apical turn, where there was a slight reduction in the number of OHCs at 1 and 10 days, which only reached statistical significance at 30 days after noise exposure (Figure 3I, Table 4). Therefore, in the middle (Figure 3A–D) and basal turns of the cochlea in noise-exposed ND-fed animals, a significant decrease in the number of OHCs was clearly present at 1 day following the noise exposure and it persisted up to 30 days post lesion (Figure 3J–K and Table 4). These results were similar to those described elsewhere [24].

#### 3.2.2. Oral Administration of ACEMg

Oral administration of ACEMg starting 10 days before the noise exposure protocol (ED-fed rats), attenuated the loss of OHCs as compared to ND-fed noise-exposed animals. Even though the average number of OHCs was also reduced in the noise-exposed ED-fed rats, such loss was significantly lower than that observed in noise-exposed ND-fed rats. In the middle (Figure 3E–H) and basal cochlear turns of ED-fed animals, the mean values of OHC survival were significantly higher than those found in the noise-exposed ND-fed group at all survival times (Figure 3J–K and Table 4). In the apical turn of the cochlea, the percentage of OHC survival, although slightly higher than in untreated noise-exposed rats, was not significantly different at 1 and 10 days after the lesion (Figure 3I, Table 4). However, at 30 days the mean values of surviving OHCs in the noise-exposed ED-fed group were significantly higher than in the noise-exposed ND-fed group, indicating greater OHC survival in ED-fed animals (Figure 3I, Table 4). These results strongly support that ACEMg improves survival of OHCs following noise overexposure.

### 3.3. Time Expression of Antioxidant Enzymes and Apoptosis Genes in the Cochlea after NIHL and ACEMg Otoprotection

#### 3.3.1. Noise-Exposed, Untreated Rats

**Figure 4:**
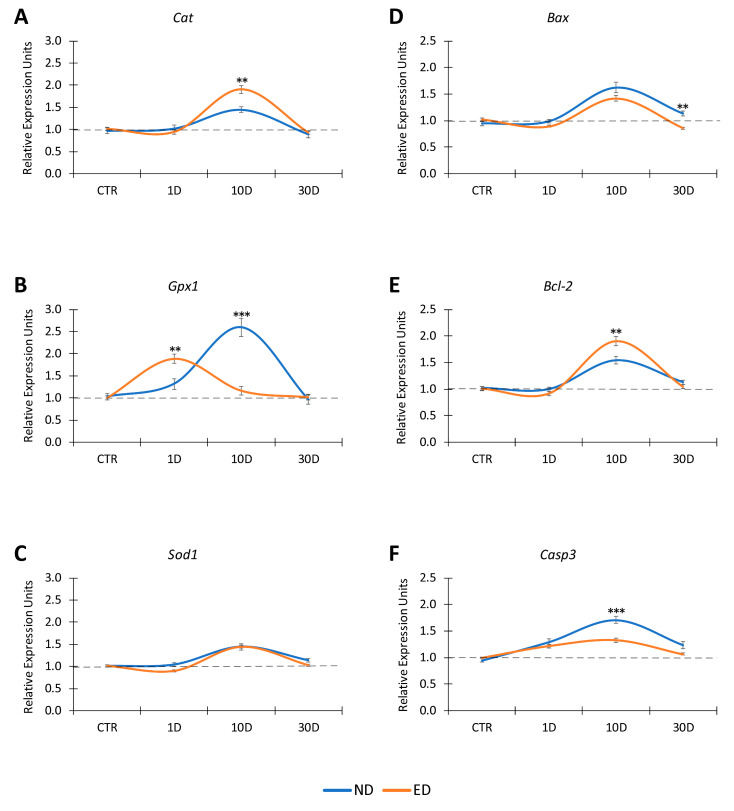
Quantitative PCR expression profiles of antioxidant enzyme (**A**–**C**) and apoptosis genes (**D**–**F**) in the cochlea of ND-fed and ED-fed animals exposed to noise. In ND-fed animals exposed to noise (blue traces in (**A**–**C**)), at day 1 after completion of noise exposure (ND-1D rats), relative expression levels of the *Cat* (**A**) and *Sod1* genes (**C**) were undistinguishable from baseline expression levels from noise-unexposed control rats (baseline). On the other hand, *Gpx1* gene expression levels were slightly, but significantly increased relative to baseline. At day 10 (ND-10D), *Cat* gene expression experienced a moderate increase, relative to baseline (**B**), and so did *Sod1* (**C**). At this same survival time, *Gpx1* gene expression was greatly increased relative to baseline (see Results section). At day 30 after noise exposure, in rats fed with ND (ND-30D) all three tested enzyme genes had returned to baseline levels. In ED-fed animals exposed to noise (orange traces in (**A**–**C**)), at day 1 after finishing noise exposure (ED-1D) *Cat* and *Sod1* gene expression were close to baseline, and undistinguishable from expression levels in ND-1D rats (**A**,**C**). In sharp contrast, *Gpx1* expression levels were significantly increased relative to ND-1D (**B**). At day 10 after noise exposure, there was a significant increase in *Cat* expression in ED-fed rats (ED-10D) relative to ND rats (ND-10D), whereas *Sod1* expression did not experience any change. On the other hand, *Gpx1* expression levels were close to baseline levels (**B**), compared with expression in ND-10. At day 30 after noise exposure (ND-30 D), gene expression levels for the three enzymes had returned to normal. Notice that oral ACEMg is linked to a relative increase in *Cat* gene expression at 10 days after noise exposure, and to a shift in the maximum expression level of *Gpx1* from day 10 to day 1 after noise exposure, compared to untreated (ND-fed) noise-exposed animals. Asterisks indicate significant differences between ND and ED rats. ∗∗ *p* < 0.01, ∗∗∗ *p* < 0.001.

The expression timelines of antioxidant enzyme genes and apoptosis-related genes in the cochlea after noise exposure, were assessed with qPCR, in search of molecular correlates of NIHL. At 1 day after finishing the noise exposure protocol, the expression levels of *Cat* (Figure 4A, Table 5) and *Sod1* (Figure 4C, Table 5) genes were undistinguishable from those of noise-unexposed control rats, which were taken as baseline expression levels.

Expression levels of the *Gpx1* gene at this survival time were around 1.3-fold higher than in unexposed animals, although such difference did not show statistical significance (Figure 4B, Table 5). In contrast, at 10 days after noise exposure significant increases in expression levels were detected for *Cat*, *Gpx1*, and *Sod1* genes. *Cat* gene expression in noise-exposed rats was near 1.5-fold relative to the unexposed condition (Figure 4A, Table 5).

The *Gpx1* gene underwent large relative overexpression, at levels over 2.5-fold higher than those found in noise-unexposed rats (Figure 4B, Table 5), whereas the expression of the *Sod1* gene was 1.5-fold above unexposed levels (Figure 4C, Table 5). Thirty days after the completion of the noise exposure protocol, gene expression of *Cat*, *Gpx1*, and *Sod1* returned to levels not significantly different from noise-unexposed rats (Figure 4A–C, Table 5).

Relative expression profiles of apoptosis genes are shown in (D, E, F). One day following noise over-exposure, there was an increase in *Casp3* expression (F) in both ND-fed (blue trace) and ED-fed (orange trace) rats. At 10 days, the expression of the three tested genes, *Bax* (D) *Bcl-2* (E) and *Casp3* (F) rose significantly in ND (blue trace) and ED (orange trace) rats as compared to the control baseline. No significant differences were observed in *Bax* expression levels (D) between ND and ED animals. However, the mean values of *Bcl-2* gene expression were significantly higher in the ED rats than in the ND rats, whereas *Casp3* (F) expression values were significantly lower than that in ND animals. At 30 days, the expression of the three tested genes diminished in ED-fed rats to values similar to control baseline whereas *Casp3* (F) and *Bax* (D) levels persisted elevated in ND rats and they were even significantly higher than in ED animals at this same survival time.

As far as apoptosis-related genes are concerned, one day after completion of the noise exposure protocol, there were no significant changes in the expression levels of the pro-apoptotic *Bax* (Figure 4D, Table 6) or the anti-apoptotic *Bcl-2* (Figure 4E, Table 6) genes. The executioner *Casp3* gene (Figure 4F, Table 6) was slightly, but not significantly, overexpressed, at levels close to 1.3-fold higher than those in unexposed animals. Ten days after noise exposure, however, all selected apoptosis genes showed significantly increased expression levels. *Bax* (Figure 4D, Table 6) and *Bcl-2* (Figure 4E, Table 6) were respectively 1.6-fold and 1.5-fold higher than in unexposed rats, whereas expression levels of the *Casp3* gene (Figure 4F, Table 6) were 1.9-fold higher. Thirty days after noise exposure, expression levels of the three apoptosis genes investigated were diminished. Expression levels of the *Bax* and *Bcl-2* genes were close to 1.1-fold higher than in unexposed rats (Figure 4D,E, Table 6). Although *Casp3* gene expression was 1.2-fold higher than in unexposed rats, the values were not significantly higher relative to unexposed controls (Figure 4F, Table 6).

#### 3.3.2. Oral Administration of ACEMg

Oral ACEMg treatment in ED-fed rats initiated10 days prior to the beginning of the acoustic overexposure protocol changed the expression pattern and timeline of antioxidant enzyme genes, compared to what was found in the noise-exposed control animals not treated with ACEMg (ND-fed group), and in noise-unexposed controls. At 1day survival, there was no significant increase in *Cat* or *Sod1* gene expression levels, in noise-exposed ED-fed animals relative to unexposed controls (Figure 4A,C, Table 5). However, ED-1D rats showed a significantly increased expression of the *Gpx1* gene of 1.9-fold relative to baseline expression levels in noise-unexposed animals and 1.4-fold higher than in ND-1D animals (Figure 4B, Table 5). At 10 days after noise exposure in noise-exposed ED-fed rats, the *Cat* gene was expressed at values close to 2.0-fold, significantly higher than those in the noise-unexposed controls, and 1.3-fold, also significantly higher than those found in ND-fed noise-exposed animals (Figure 4A, Table 5). In turn, at this survival time of 10 days, *Gpx1* gene expression levels returned to values closer to the control condition, with relative expression levels around 1.2-fold (Figure 4B, Table 5). Even though the expression of the *Sod1* gene was 1.4-fold, significantly higher compared to the noise-unexposed rats, the values were not significantly different to those found in ND-fed noise-exposed animals at the same survival time (see above) (Figure 4C, Table 5). Similar to ND-fed noise-exposed rats, at 30 days after the noise exposure, the expression levels of the three antioxidant enzyme genes tested in the animals fed with ACEMg decreased to values close to those in control unexposed animals (Figure 4A–C, Table 5).

Regarding genes involved in apoptosis regulation, at 1 day after the noise exposure protocol, the expression levels of *Bax*, *Bcl-2*, and *Casp3* genes in ACEMg-treated animals, ED-fed, were similar to those found at the same post-exposure time after noise in the ND-fed animals (Figure 4D–F, Table 6). At 10 days after noise exposure in ED-fed rats, the pro-apoptotic *Bax* gene, was expressed at values of 1.4-fold, which were significantly higher than those in the noise-unexposed rats (Figure 4D, Table 6). The anti-apoptotic *Bcl-2* gene, on the other hand, was expressed at values close to 2-fold that were significantly higher than in the noise-unexposed control rats, and also significantly higher than the expression values detected at the same time point after noise overstimulation in the ND-fed animals (Figure 4E, Table 6). *Casp3* gene expression levels in ED-fed animals, although still significantly higher than the noise-unexposed controls were significantly decreased compared to the ND-fed unexposed rats at the same survival time (Figure 4F, Table 6). At day 30 after noise exposure and oral ACEMg administration, the expression for the three tested genes involved in apoptosis regulation had returned to values similar to normal, noise-unexposed animals (Figure 4D–F, Table 6). When compared to the ND-30D fed rats, only the *Bax* gene showed significant differences, its expression level being lower in the ED-30D group (Figure 4D, Table 6).

### 3.4. Distribution of Immunostaining for Antioxidant Enzymes and Apoptosis-Related Proteins in the Cochlea after NIHL and ACEMg Otoprotection

#### 3.4.1. Noise-Exposed, Untreated Rats

**Figure 5:**
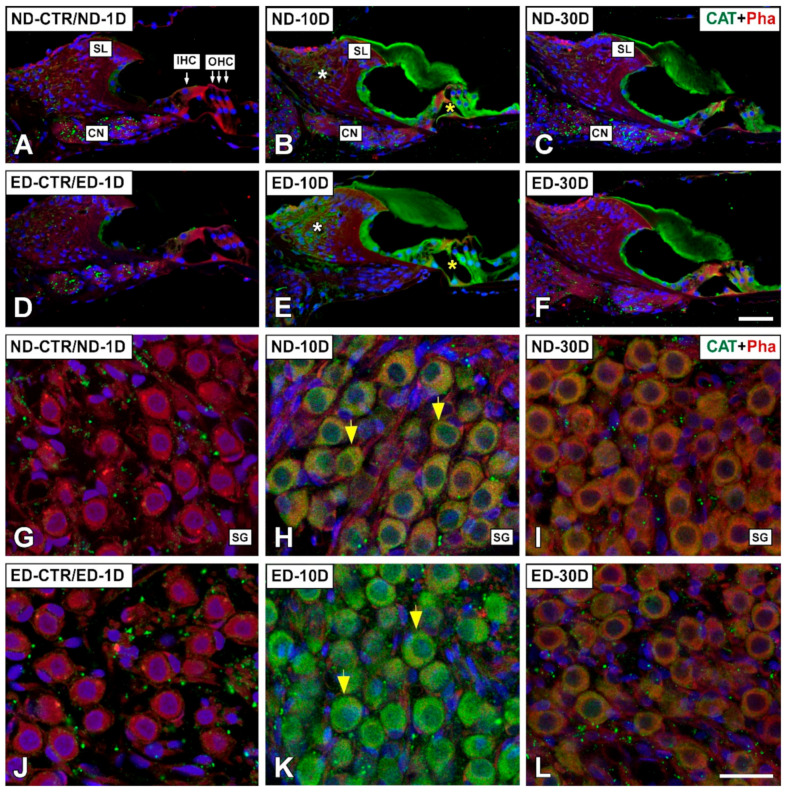
Laser confocal microscopy images showing the distribution of CAT immunolabeling in the cochlea of ND and ED-fed rats at 1D, 10D, and 30D after noise-exposure. CAT immunofluorescence is green. Red fluorescence is phalloidin labeling. When compared with the untreated controls (ND-CTR) and the ND-1D group ((**A**,**G**) shows an example from a ND-1D animal), under identical antibody incubation conditions CAT-immunostaining was more intense in the organ of Corti (yellow asterisk in B), the spiral limbus (white asterisk in (**B**)), and the spiral ganglion (yellow arrows in (**H**)) at 10D post-exposure. The immunostaining intensity was decreased at day 30 after noise exposure although it was still evident in the organ of Corti (**C**) and the spiral ganglion (**I**). Following oral administration of ACEMg, immunostaining increased in the noise-exposed cochlea at 10D post-exposure (**E**,**K**) when compared to non-treated animals at the same survival time and the ND-1D group (**D**,**J**). Note that at 30D post-exposure, the staining intensity between treated (**C**,**I**) and untreated (**F**,**L**) rats was comparable. Abbreviations: CN, cochlear nerve; IHC, inner hair cell; OHC, outer hair cells; SL, spiral limbus; SG, spiral ganglion. Scale bar = 50 µm in F and 25 µm in (L).

**Figure 6:**
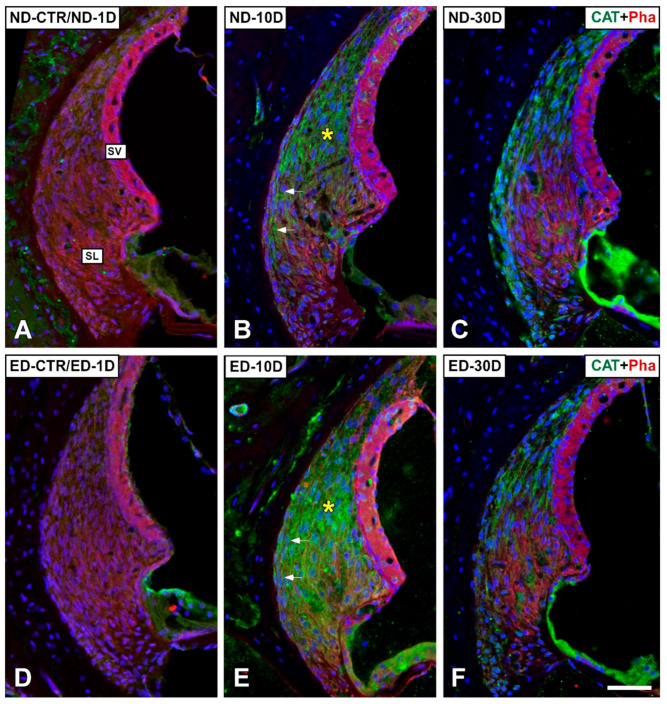
Confocal images showing the distribution of CAT immunolabeling in the lateral wall of ND and ED-fed rats at 1D, 10D, and 30D after noise-exposure. CAT immunolabeling is green. Red is phalloidin staining. When compared with the control, untreated condition and the ND-1D group (**A**), at 10D post-exposure, immunostaining for CAT was particularly intense in the type I/III fibrocyte region of the spiral ligament (asterisk and arrows in (**B**)). Oral administration of ACEMg resulted in an increased CAT immunolabeling (arrows and asterisk in (**E**)) relative to untreated animals (**D**). At longer survival times (30D post-exposure), there were no apparent differences between untreated (**C**) and treated (**F**) rats. Abbreviations: SL, spiral ligament; SV, stria vascularis. Scale bar = 50 µm is shown in F.

**Figure 7:**
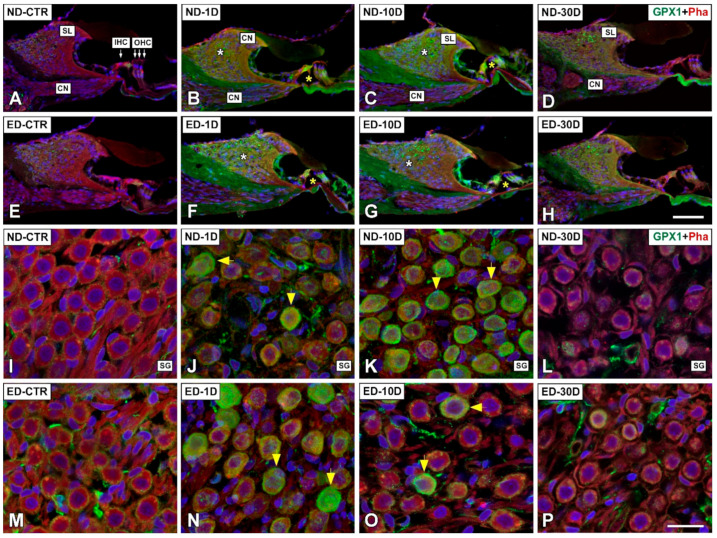
Confocal microscopy images showing the distribution of GPX1 in the cochlea of ND and ED-fed rats at 1D, 10D, and 30D after noise exposure. Green immunofluorescence is GPX1. Red fluorescence is phalloidin staining. Compared with the control untreated condition (**A,I**), at 1D post-noise exposure there was a slight increase in GPX1-immunostaining intensity in the organ of Corti (yellow asterisk in (**B**)), the spiral limbus (white asterisk in (B)) and the spiral ganglion (yellow arrows in (**J**)). At day 10 after noise exposure, immunostaining intensity increased in the spiral limbus (white asterisk in (**C)**), the organ of Corti (yellow asterisk in (**C**))) and the spiral ganglion (arrows in (**K**)) but at later survival times (30D post-exposure), it was minimal (**D**,**L**). ACEMg treatment resulted in increased immunostaining at 1D after the noise exposure that was particularly evident in the spiral limbus (white asterisk in (**F**)) and in the spiral ganglion (yellow arrows in (**N**)), compared with untreated animals at the same survival time (**B**,**J**) and the ED-CTR group (**E**,**M**). At 10D post-noise exposure, however, ACEMg treatment resulted in decreased immunostaining in the organ of Corti, the spiral limbus and the spiral ganglion (**G**,**O**) when compared with the ND-10D group (**C**,**K**). At 30D post-exposure, there were no evident differences between untreated (**D**,**L**) and treated (**H**,**P**) rats. Abbreviations: CN, cochlear nerve; IHC, inner hair cell; OHC, outer hair cells; SL, spiral limbus; SG, spiral ganglion. Scale bar = 50 µm in H and 25 µm in P.

**Figure 8:**
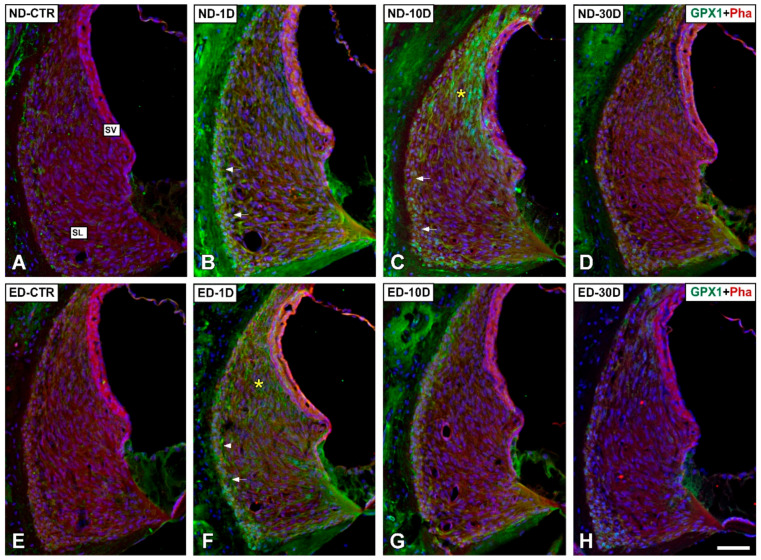
Confocal microscopy images showing the distribution of GPX1 in lateral wall of ND and ED-fed rats at 1D, 10D, and 30D after noise exposure. Green is GPX1 immunolabeling. Red is phalloidin labeling. Compared with the control untreated condition (**A**), at day 1 post-exposure, immunostaining for GPX1 increased slightly in the spiral ligament (arrows in (**B**)). Visually, immunostaining intensity peaked at day 10 post-exposure (asterisk and arrows in (**C**)) and returned to normal at day 30 (**D**). Oral administration of ACEMg, resulted increased GPX1 immunolabeling (arrows and asterisk in (**F**)) at day 1 post-noise exposure as compared with the control conditions (**A**,**E**) and the ND-1D (arrows in (**B**)) group. Immunostaining intensity decreased by day 10 (**G**) and was virtually absent at day 30 (**H**). Abbreviations: SL, spiral ligament; SV, stria vascularis. Scale bar = 50 µm is shown in H.

**Figure 9:**
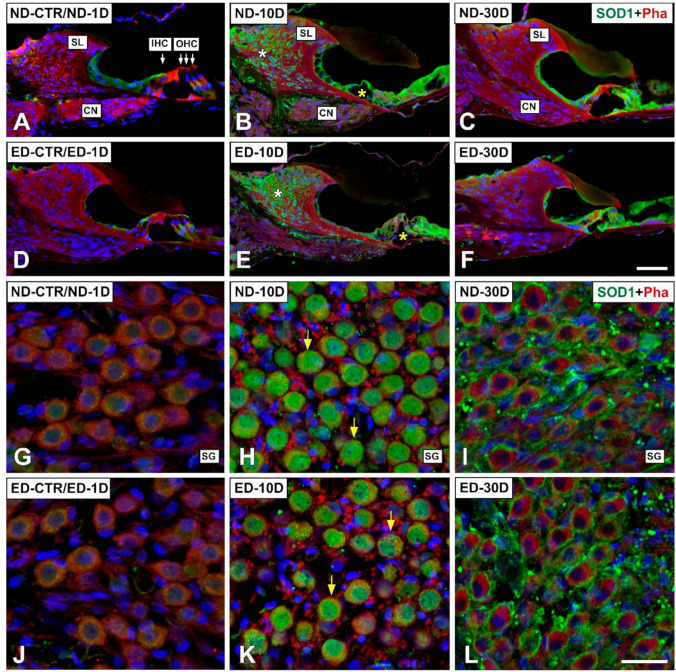
Confocal microscopy images showing the distribution of SOD1 in the cochlea of ND and ED-fed rats at 1D, 10D, and 30D after noise-exposure. Green fluorescence is SOD1 immunolabeling. Red fluorescence is phalloidin staining. Compared with the control untreated condition and the ND-1D group ((**A**,**G**) shows an example from a ND-CTR animal), at day 10 post-noise exposure there was an increase in SOD1-immunostaining intensity in the organ of Corti (yellow asterisk in (**B**)), the spiral limbus (white asterisk in (**B**)) and the spiral ganglion (yellow arrows in (**H**)). At 30 days after noise-exposure, immunostaining intensity decreased, although it was still evident in the organ of Corti (**C**) and the spiral ganglion (**I**). Following oral administration of ACEMg, there were no differences among untreated and treated groups for any of the cochlear structures analyzed at any of the survival times evaluated (compare (**A**–**C**) with (**D**–**F**); and (**G**–**I**) with (**J**–**L**)). Abbreviations: CN, cochlear nerve; IHC, inner hair cell; OHC, outer hair cells; SL, spiral limbus; SG, spiral ganglion. Scale bar = 50 µm in F and 25 µm in L.

**Figure 10:**
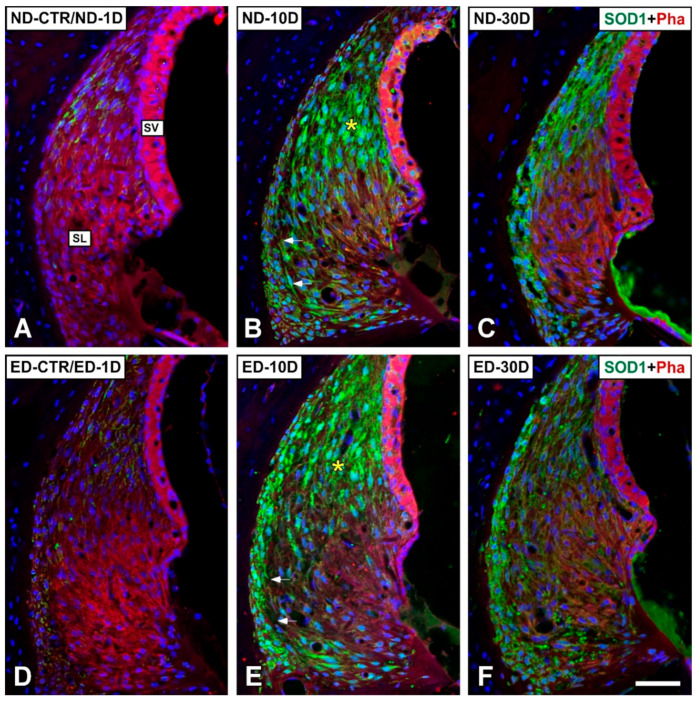
Confocal microscopy images showing the distribution of SOD1 in the lateral wall of ND and ED rats at 1D, 10D, and 30D after noise exposure**.** Green immunofluorescence is SOD1. Red fluorescence is phalloidin staining. When compared with the ND-CTR and the ND-1D groups (**A**), at 10 days post-exposure, immunostaining for SOD1 was particularly intense in the type I/III fibrocyte region of the spiral ligament (asterisk and arrows in (**B**)). At longer post-noise exposure survival times (30D post-exposure), SOD1 immunostaining decreased (**C**) when compared with the ND-10D group (**B**). Oral administration of ACEMg, resulted in no differences among untreated and treated groups (compare (**A**–**C**) with (**D**–**F**)). Abbreviations: SL, spiral ligament; SV, stria vascularis. Scale bar= 50 µm in (**F**).

The distribution of immunostaining for the antioxidant enzymes, CAT (Figure 5 and Figure 6), GPX1 (Figure 7 and Figure 8), and SOD1 (Figure 9 and Figure 10) was first examined in the cochlea of noise-exposed and unexposed untreated rats. Homogeneous antibody incubation conditions allowed evaluation of visual differences in immunolabeling intensities for the same antibody. In the unexposed cochlea, immunostaining for these enzymes was weak in the organ of Corti, the spiral limbus, the spiral ganglion, and the spiral ligament. At one day after noise exposure, the immunostaining for CAT (Figure 5A,G and Figure 6A) and SOD1 (Figure 9A,G and Figure 10A) in all the above-mentioned cochlear structures was also weak, and similar to the unexposed cochlea. However, immunostaining for GPX1 was slightly more intense in the spiral limbus (white asterisk in Figure 7B), the organ of Corti (yellow asterisk Figure 7B), and the spiral ligament (arrows in Figure 8B), when compared to the unexposed cochlea (Figure 7A and Figure 8A). Also, scattered immunolabeled neurons were observed at this survival time in the spiral ganglion (yellow arrows in Figure 7J). At day 10 post-exposure, immunostaining intensities for GPX1 C,K and Figure 8C) and to a lesser extent for CAT (Figure 5B,H and Figure 6B) and SOD1 (Figure 9B,H and Figure 10B) were increased in all cochlear structures evaluated. Notably, CAT (asterisk and arrows in Figure 6B), GPX1 (asterisk and arrows in Figure 8C) and SOD1 (asterisk and arrows in Figure 10B) -stained fibrocytes were identified in the spiral ligament. Based on previous studies [33,40,41], they were mostly characterized as type I and III. At the latest time point assessed (30 days), immunostaining for all three antioxidant enzymes in all the above-mentioned cochlear structures was decreased in the noise-exposed cochlea (Figure 5C,I and Figure 6C for CAT; Figure 7D,L, and Figure 8D for GPX1; Figure 9C,I, and Figure 10C for SOD1) but it still remained elevated relative to unexposed controls.

**Figure 11:**
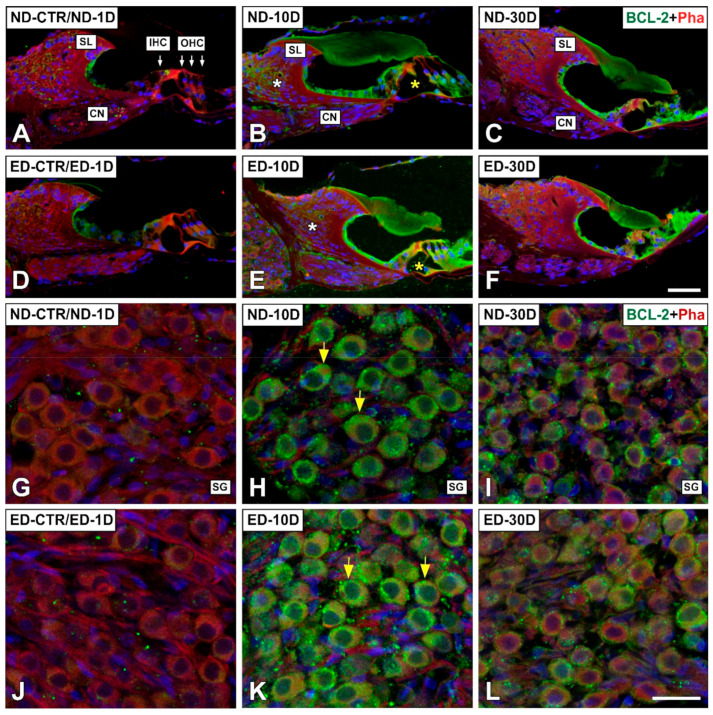
Confocal microscopy images showing the distribution of BCL-2 in the cochlea of ND and ED-fed rats at 1D, 10D, and 30D after noise exposure. Green immunofluorescence is BCL-2. Red fluorescence is phalloidin staining. Compared with the control untreated condition and the ND-1D group (**A**,**G**), there was an increase in BCL-2-immunostaining in the organ of Corti (yellow asterisk in (**B**)), the spiral limbus (white asterisk in (**B**)) and the spiral ganglion (yellow arrows in (**H**)) at 10D post-exposure. Note that immunostaining intensity decreased at day 30 after noise exposure, although it was still present in the organ of Corti (C) and the spiral ganglion (**I**). At 10 days after the noise exposure, ACEMg treatment resulted in increased immunostaining intensity (E,K) when compared with non-treated animals at the same survival time (B,H) and the noise unexposed control and ND-1D groups (**D**,**J**). Note that at 30 days post-exposure, immunostaining intensities between treated (**F**,**L**) and untreated (**C**,**I**) rats were not visually different. Abbreviations: CN, cochlear nerve; IHC, inner hair cell; OHC, outer hair cells; SL, spiral limbus; SG, spiral ganglion. Scale bar = 50 µm in (F) and 25 µm in (**L**).

**Figure 12:**
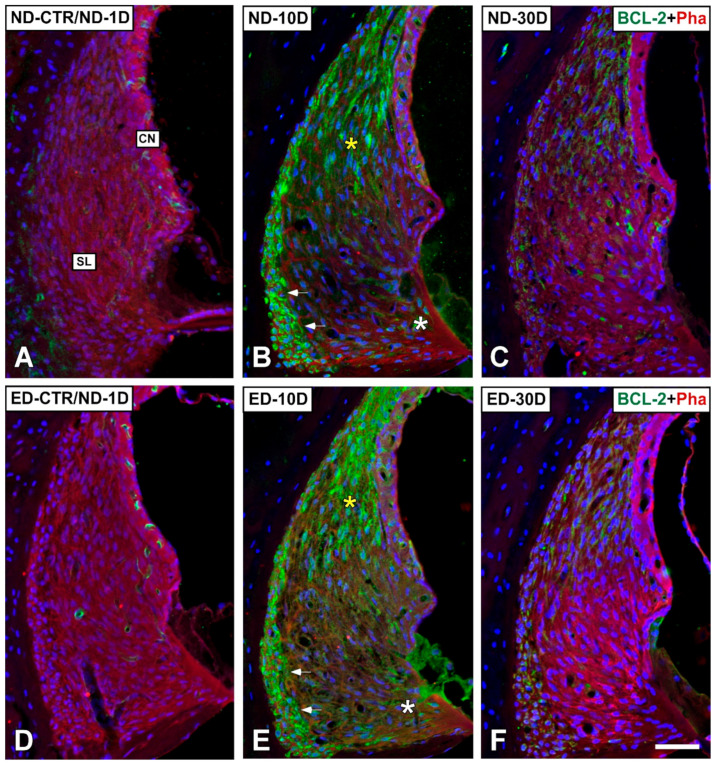
Confocal microscopy images showing he distribution of BCL-2 in the lateral wall of ND and ED-fed rats at 1D, 10D, and 30D after noise-exposure. Green immunofluorescence is BCL-2. Red fluorescence is phalloidin staining. Compared with the control untreated condition and the ND-1D group (**A**), at 10D post-noise exposure immunostaining for BCL-2 was particularly strong in the area where type I/III fibrocytes of the spiral ligament are located (yellow asterisk and arrows in (**B**)). Oral administration of ACEMg, resulted in increased immunostaining (arrows and asterisk in (**E**)) when compared either with control or 1D untreated and treated (**D**) animals (yellow asterisk and arrows in (**B**)). Note that oral administration of ACEMg at this survival time also led to an increase in type IV BCL-2-immunostained fibrocytes, compared with the ND-10D group (white asterisks in (**B**) and (**E**)). At longer survival times (30D post-exposure), there were no apparent differences between untreated (**C**) and treated (**F**) rats. Abbreviations: SL, spiral ligament; SV, stria vascularis. Scale bar = 50 µm in (**F**).

Regarding apoptosis-related proteins, the expression for BCL-2 was evaluated. In our hands, other antibodies did not give consistent immunolabeling differences across experimental conditions. At day 1 after noise exposure, there were not differences in immunostaining relative to the control noise-unexposed condition (Figure 11A,G and Figure 12A). At day 10, however, immunostaining was increased in the organ of Corti (yellow asterisk in Figure 11B), the spiral limbus (white asterisk in Figure 11B), the spiral ganglion (yellow arrows in Figure 11H), and the spiral ligament, particularly in areas were type I and III fibrocytes are located, as compared to unexposed rats (yellow asterisk and arrows in Figure 12B). At day 30, BCL-2 immunostaining decreased although it was still evident in the Organ of Corti (Figure 11C) and the spiral ganglion (Figure 11I).

#### 3.4.2. Oral Administration of ACEMg

At 1 day after the noise exposure, ACEMg treatment in ED-fed animals, resulted in no apparent changes in the immunostaining for CAT (Figure 5D,J and Figure 6D) and SOD1 (Figure 9D,J and Figure 10D) in the cochlea relative to untreated, noise exposed animals. However, immunostaining for GPX1 was increased, particularly in the spiral limbus (white asterisk in Figure 7F), the spiral ganglion (yellow arrows in Figure 7N), and the spiral ligament (yellow arrows in Figure 8F) as compared to exposed non-treated animals (Figure 7 and Figure 8). At 10 days post-exposure in treated animals, there was an increase in immunostaining for CAT (Figure 5E,K and Figure 6E) as well as a decrease for GPX1 (Figure 7G,O and Figure 8G) in the cochlear structures evaluated. At longer time points after noise exposure, there was a decrease in immunostaining intensity for either CAT (Figure 5F,L and Figure 6F) or GPX1 (Figure 7H,P and Figure 8H) staining in ACEMg-treated rats that was particularly evident in the organ of Corti, the spiral ganglion and the spiral ligament. Similar to the gene expression data, no visible modifications in SOD1 staining in the noise-exposed cochlea were observed after ACEMg treatment (Figure 9 and Figure 10).

Regarding BCL-2 immunostaining in ACEMg treated (ED-fed) animals, there was an increase at day 10 after noise-exposure mostly in the organ of Corti (yellow asterisk in Figure 11E), the spiral ganglion (yellow arrows in Figure 11K) and the spiral ligament (yellow asterisk and arrows in Figure 12E). An increase in immunolabeled type IV fibrocytes was noticeable in the spiral ligament in the ED-10D (white asterisk in Figure 12E) group when compared to ND-10D (white asterisk in Figure 12B) animals. However, at day 30 there were no apparent differences between noise-exposed treated and untreated animals (Figure 11F,L and Figure 12F).

## 4. Discussion

We report that a combination of antioxidant vitamins (A, C, and E) and Mg^2+^ (ACEMg) administered orally in rats significantly limits NIHL. Such otoprotection of ACEMg against noise damage involves expression regulation of antioxidant enzymes and proteins involved in apoptosis.

In rats, oral ACEMg significantly reduced auditory threshold degradation after NIHL, with an overall preference for frequencies between 2 and 8 kHz. Although auditory thresholds still were relatively high after treatment, shifts in threshold were much narrower compared to animals which did not receive oral ACEMg. This suggests a significant otoprotective effect of oral ACEMg against NIHL. This is also supported by a previous proof of concept study in guinea pigs, in which vitamins A, C, E, and MgSO_4_ were injected intraperitoneally after a permanent threshold shift NIHL [20]. It was proposed that antioxidant vitamins and Mg^2+^ act synergistically to protect against NIHL, combining the antioxidant properties of vitamins A, C, E, and the cochlear vasodilation power of Mg^2+^. When injected together, threshold shifts were much more restricted, in the range of 8–23 dBs higher than normal, depending on the stimulus frequency, compared to the 32 to above 50 dB shifts obtained with separate administration of vitamins or Mg^2+^. Differences in threshold recovery values reported by these authors in comparison with those reported here probably relate to different administration routes. Oral ACEMg likely provides lower bioavailability, but still sufficient for a robust otoprotective effect. In fact, a diet supplemented with vitamins A, C, E, and Mg^2+^ also reduced significantly threshold shifts in ABRs in CBA/J mice with permanent NIHL [42]. Reductions of 15–20 dBs were reported, mainly in the 10–20 kHz frequency range, when diet supplementation was initiated 28 days before induction of NIHL. Our findings showed comparable threshold recoveries with oral ACEMg in rats, initiated as shortly as 10 days before induction of NIHL. However, we also observed a trend towards significant threshold recoveries at wider frequency ranges, encompassing 8 kHz and lower, not reported previously [22,42]. This may be related to different susceptibilities among species, differences in the concentration of vitamins and Mg^2+^ in the supplemented chow, differences in bioavailability or pharmacokinetics or a combination thereof. Also, the time window between initiation of oral ACEMg administration and exposure to noise may be of relevance for otoprotection. Again, evidence from mice [42] and from rats in the present study support that oral administration of ACEMg from 28 days to as early as 10 days before noise exposure results in antioxidant concentrations with effective otoprotection against noise Whether antioxidant administration closer to or coincident with the time of noise-exposure will show comparable otoprotection will be a function of the time taken to obtain sufficient antioxidant concentrations in relation to the irreversibility of cellular damage. This is currently unknown, and important to determine for potential therapeutical applications.

We then sought to unravel cellular and molecular events related to ACEMg-mediated threshold recovery and otoprotection. As expected, hair cell counts after the NIHL protocol showed progressive and extensive OHC loss. As shown previously, OHC loss increased at longer survival times after noise exposure (10 days and 30 days) and it was localized mostly in the middle and basal turns [24,31]. After ACEMg treatment, in ED-fed rats, the percentage of surviving OHCs was significantly higher than in non-treated, ND-fed animals, at all survival times after the exposure. In guinea pigs receiving daily intraperitoneal injections of ACEMg prior to exposure to octave-band noise (4 kHz, 120 dB SPL) for 5 h, there was a reduction of OHC death even when the injection was administered as short as 1 h before the exposure [20]. This protective effect against noise induced OHC loss, however, was not detected in CBA/J mice. Threshold recovery was attributed to antioxidant protection of surviving OHCs potentiated by improved structure and therefore, function of the spiral ligament and stria vascularis [42]. Oral ACEMg in higher dietary amounts has been shown to induce modest recovery of the number of inner hair cells in the Gjb2-CKO mouse, a model of a frequent form human hereditary deafness [22]. Other otoprotective compounds such as HK-2 (1-(5-hydroxypyrimidin-2-yl) pyrrolidine-2,5-dione), a redox modulating drug known to reduce oxidative stress, also has been demonstrated to prevent NIHL. In this regard, oral administration of HK-2 to Sprague Dawley rats exposed to 8–16 kHz octave band noise presented for 8 h/d for 21 days at 95 dB SPL, not only limited hair cell loss but also significantly reduced oxidative stress [43]. Altogether, these results support that the administration route, dosage, and species susceptibility, along with survival time after the exposure dictate targets of drug otoprotection. High enough doses and/or bioavailability of ACEMg seem to limit loss of hair cells, mainly OHC. Lower doses have a limited ability to protect hair cells from death. However, protection from noise still is present, probably by limiting oxidative stress damage in the surviving hair cells and, very importantly, in lateral wall structures in charge of ion homeostasis [42], key for the generation of the endocochlear potential along with an ionic microenvironment adequate for the survival of the neuroepithelium [43], as discussed further in detail.

Antioxidant vitamin intake likely potentiates the free radical scavenging effects of those naturally active in cells. For example, it is known that beta-carotenoids, as vitamin A precursors, prevent lipid peroxidation and scavenge highly reactive singlet oxygen [44]. Vitamin E reduces peroxyl radicals in cell membranes whereas vitamin C eliminates free radicals in aqueous phase and contributes to regenerating oxidized vitamin E [45]. This, in combination with cochlear vasodilation and probably excitotoxicity protection induced by Mg^2+^, generates synergistic interactions which may be at the core of the otoprotective mechanisms [20]. However, we found that ACEMg also influences, directly or indirectly, enzymatic antioxidant defenses by regulating the expression of key antioxidant enzymes, catalase, and glutathione peroxidase 1, with no apparent effects on others such as superoxide dismutase.

Firstly, it seems that there is a characteristic expression timeline of these enzyme genes in the cochlea after NIHL. Although, by itself, the level of free radicals is a feedback regulator of antioxidant enzymes, additional layers of regulation may exist [46,47]. Actually, fast downregulation of antioxidant enzyme genes takes place shortly after initiating noise exposure (data not shown). These suggests that, in spite of free radical buildup, enzyme-mediated antioxidation mechanisms may become partially ‘exhausted’ in the first stages of noise overexposure. In this regard, it has also been reported that CAT levels drop immediately after exposure to noise leading to permanent threshold shift [46]. In the present study, at 1 day after finishing noise exposure, *Cat* and *Sod1* gene expression was undistinguishable from normal values, whereas *Gpx1* was slightly but not significantly upregulated. Later, at 10 days after noise exposure, the *Gpx1* gene underwent significant, particularly large upregulation, alongside *Cat* and *Sod1* which were also upregulated, although at lower levels. Upregulation of the *Gpx1* gene between day 1 and day 10 after noise exposure suggests that this enzyme may articulate early cochlear enzyme-mediated antioxidation responses to noise damage. It has been shown that targeted deletion of the *Gpx1* gene in mice, dramatically increases NIHL [47]. Changes in *Cat* levels also have been found to be related to protection against NIHL [46]. *Sod1*, however, seems to relate in a more complex way with noise damage in the cochlea. *Sod1* gene knock-out mice show just slightly more susceptibility to NIHL than wild type mice, at least at young ages [48,49].

*Sod1* overexpression, on the other hand, does not protect against NIHL [50] or even increases the effects of noise trauma [51]. It is likely that excess H_2_O_2_ produced by increased enzymatic levels of SOD1 may damage cells if SOD1 activity is not balanced by GPX1 [50]. Therefore, the interplay among *Gpx1*, *Cat*, and *Sod1* gene expression levels at day 10 after noise exposure probably reflects a mounting integrated antioxidant enzymatic response, which may be construed as an attempt to limit the consequences of noise damage to the cochlea. Finally, at 30 days after noise exposure, *Gpx1*, *Cat*, and *Sod1* gene expression returned to normal. Corroborating and expanding results on gene expression, immunocytochemistry shows increased intensity of antioxidant enzyme immunolabeling and therefore a, likely protective, increase in antioxidant response after NIHL, located mainly in the organ of Corti and the spiral ligament and also in the spiral limbus and the spiral ganglion as reported previously [52,53].

Secondly, we found that ACEMg treatment profoundly influences the expression timeline of antioxidant enzyme genes after NIHL. As already mentioned, vitamins A, C, and E scavenge or chain-break ROS/RNS [54], which along with the additive/synergistic vasodilation, and probably also anti-excitotoxic effects of Mg^2+^, are essential for otoprotection [20,42]. However, they also seem to have less well understood roles in regulating gene expression [19], which may include antioxidant enzymes [55,56]. We found that after oral ACEMg treatment, initiated 10 days before noise exposure, there is an ‘accelerated’ antioxidant enzyme gene expression response, relative to untreated NIHL. Actually, at day 1 after noise exposure, patterns of gene expression following oral administration of ACEMg are characterized by significantly increased levels of *Gpx1*, similar to those seen at day 10 after NIHL with no antioxidant treatment, whereas *Cat* and *Sod1* expression is not affected by treatment, relative to their expression levels after NIHL. Interestingly, at day 10 after noise exposure following ACEMg administration, expression levels of *Gpx1* dropped close to normal baseline, whereas the *Cat* gene was upregulated. In other words, we hypothesize that ACEMg oral treatment may increase the limited efficiency of the naturally occurring enzymatic antioxidant response taking place in the cochlea after NIHL, by inducing a much earlier peak in the expression of *Gpx1* relative to untreated NIHL, as seen at day 1 after treatment. At day 10, *Gpx1*expression levels dropped closer to baseline, whereas *Cat* levels were significantly increased relative to levels seen in NIHL. Therefore, in ND-fed rats, not treated with ACEMg, there is a monophasic expression profile of antioxidant enzymes after NIHL, characterized by an increased late expression of *Gpx1*, *Cat*, and *Sod1* genes, seen at 10 days after noise overstimulation. Following ACEMg treatment, there is a change to a biphasic expression profile, with an earlier increase in *Gpx1* levels, as detected at day 1, and increased *Cat* levels predominating at day 10 after treatment. *Sod1* levels are unaffected by treatment, maintaining increased levels of expression at day 10. This expression pattern, as far as it correlates with enzyme activity, seemingly limits NIHL more efficiently, because the ACEMg-related increase in enzyme expression levels starts earlier, at the expense of increased *Gpx1*, when noise damage to the cochlea still is, at least in part, reversible [5], and is maintained later in time at the expense of increased *Cat* levels, along with *Sod1*. Immunocytochemical labeling for these antioxidant enzymes also supports and expands this notion, showing that ACEMg attenuates oxidative stress on cochlear structures that are known to be susceptible to noise-induced permanent damage [46,57]. Also, the distribution of immunolabeling for antioxidant enzymes highlights the role of ‘non-sensory’ cochlear structures, such as the inner spiral limbus and the spiral ligament, in natural or induced protective responses to noise overexposure. Changes in the expression of antioxidant enzymes in the inner spiral limbus may regulate local responses to noise by limiting oxidative stress in connective tissue cells in this structure, likely involved in cochlear inflammatory-immune responses [58]. On the other hand, changes in antioxidant enzyme immunolabeling in fibrocyte populations in the spiral ligament support an important role for these cells in cochlear protection against noise. Limiting oxidative stress in these cells may assist in preserving their function in local K+ clearance and recycling pathways, essential to maintain the endocochlear potential, necessary for the normal function of the receptor [59].

Oxidative stress products are major mediators of apoptotic cell death [11,60]. We found that, in the cochlea, key apoptosis genes change their expression patterns after NIHL, and that such changes are influenced by ACEMg treatment, compatible with promotion of cell survival. We tested by qPCR, the expression of two key members of the *Bcl-2* family of apoptosis genes, *Bax* and *Bcl-2*. The BAX protein critically contributes to apoptosis by assembling pores in the outer mitochondrial membrane, which increase permeability to apoptosis inducers [61,62]. BCL-2, on the other hand, has key anti-apoptotic roles by antagonizing the effects of several pro-apoptotic proteins, which includes blockade of BAX oligomerization, thus interfering with the assembly of mitochondrial membrane permeabilization pores [61,62]. There seems to be a delicate balance between both proteins, which is one specific aspect of the overall delicate regulation of apoptosis mechanisms [63]. We also looked at the expression of *Casp3*, a main executioner caspase involved in the last stages of apoptotic breakdown [63]. In spite of the complex array of molecules and pathways involved in apoptosis, the expression timeline of these three proteins after NIHL and ACEMg administration, provides insights on otoprotection mechanisms. Our results show that at one day after the end of noise exposure, both *Bax* and *Bcl-2* gene expression were similar to normal levels whereas *Casp3* significantly increases, supporting activation of apoptotic events. Ten days after noise exposure, both *Bax* and *Bcl-2* gene expression levels were significantly increased relative to control rats and this coincides also with highly increased *Casp3* expression, suggesting active apoptosis. Thirty days after the end of noise exposure, *Bax* and *Bcl-2* expression returned to values close to the control condition, while *Casp3*, although at lower levels than at day 10 was still statistically significantly increased compared to control values. This may indicate a trend towards progressive return to a normal apoptosis rate past day 30 after noise exposure, which matches stabilization of hair cell loss. Changes in expression levels of *Bax* and *Bcl-2* are closely matched at different noise exposure times. In this regard, increased *Bcl-2* expression apparently is not enough to counteract increased apoptosis rates during NIHL. In coincidence with increased gene expression levels, immunocytochemistry shows that BCL-2 immunostaining intensity progressively increases in the cochlea between day 1 and day 10 after exposure. In agreement with previous reports [52,53], the immunolabeling for BCL2 was concentrated in the organ of Corti, the spiral limbus, and the spiral ligament along with the spiral ganglion, supporting increased apoptotic processes in these regions after NIHL.

After oral administration of ACEMg, we found changes in the expression patterns of *Bcl-2* and *Casp3*, compared to those found after NIHL, suggesting that this otoprotective combination directly or indirectly modulates apoptotic events. The most dramatic changes are seen at day 10 after noise exposure, with ACEMg intake initiated 10 days before sound overstimulation. At this time, whereas *Bax* expression levels were just slightly lower than those found at 10 days post-noise exposure with no treatment, *Bcl-2* expression levels were significantly increased relative to untreated NIHL. Comparatively higher levels of anti-apoptotic *Bcl-2* expression 10 days after noise exposure attained with ACEMg treatment may counteract increased apoptosis rates after NIHL. Also, *Casp3* expression levels were significantly decreased by ACEMg treatment, suggesting that this may be part of mechanisms downstream of the regulation of antioxidant enzymes by which ACEMg exerts its otoprotective effects, ultimately preserving hair cell integrity and function.

In summary, we have found that oral ACEMg exerts a very significant preservation of hearing in a rat model of NIHL. Such ACEMg otoprotection against noise seems to involve potentiation in time of antioxidant enzyme expression levels and regulation of anti-apoptotic proteins such as BCL-2 and CASP3. These findings add to mechanistic knowledge on antioxidant otoprotection which may help to optimize strategies for translation into treatments for NIHL in humans.

## Figures and Tables

**Table 1:** Oligonucleotides and qPCR settings.

Gene	Accesion No.	Sequence (5’–3’)	Genomic Location (Exons; FW–RV)	Bp	PCR Efficiency	R^2^
*Hprt1*	NM_012583.2	FW:TCCCAGCGTCGTGATTAGTGA RV:CCTTCATGACATCTCGAGCAAG	1/2–3 ^a^	152	97.3%	0.9996
*Tbp*	NM_001004198.1	FW:CCCACATCACTGTTTCATGG RV:CCGTAAGGCATCATTGGACT	1/2–3	215	99.2%	0.9995
*Bax*	NM_017059	FW: CGAGCTGATCAGAACCATCA RV:CTCAGCCCATCTTCTTCCAG	5–6	91	98.4%	0.9994
*Bcl-2*	NM_016993	FW:GAGCGTCAACAGGGAGATGT RV:CTCACTTGTGGCCCAGGTAT	1–2	242	99.3%	1.000
*Casp3*	NM_012922	FW:GGCCCTGAAATACGAAGTCA RV:GGCAGTAGTCGCCTCTGAAG	4–5	209	97.6%	0.9986
*Cat*	NM_012520	FW:GAGGAAACGCCTGTGTGAGA RV:TTGGCAGCTATGTGAGAGCC	11–13	201	98.8%	0.9997
*Gpx1*	NM_030826	FW:GTTTCCCGTGCAATCAGTTC RV:CATTCCGCAGGAAGGTAAAG	1–2	71	99.3%	0.9972
*Sod1*	NM_017050	FW:CCACTGCAGGACCTCATTTT RV:CACCTTTGCCCAAGTCATCT	3–5	216	99.1%	0.9991

^a^—Primers that match on an exon–exon junction; R^2^—Regression coefficient; Bp: Product size**.** Primer pairs were designed using the specific softwarePrimer3 Plus, freely available at: http://www.bioinformatics.nl/cgi-bin/primer3plus/primer3plus.cgi/.

**Table 2:** Antibodies used for immunohistochemistry.

Primary Antibody	Immunogen	Host	Code/Clone	Dilution	Manufacturer
Catalase	C-terminus of catalase of mouse origin	Goat	SC-34285	1:100	Santacruz, Biotechnology, Inc., Dallas, TX, USA
GPX1	Synthetic peptide conjugated to KLH derived from within residues 150 to the C-terminus of Human GPX1	Rabbit	ab22604	1:100	Abcam plc. Cambridge, UK
SOD1	C-terminus of SOD-1 of human origin	Goat	SC-8637	1:100	Santacruz, Biotechnology
Bcl-2	N-terminus of Bcl-2 of human origin	Goat	SC-492 (N19)	1:100	Santacruz, Biotechnology

**Table 3:** Mean ± SE and ANOVA of the interaction between diet, noise over-exposure, and auditory thresholds and threshold d shifts.

Groups		Frequencies (kHz)						
		0.5	1	2	4	8	16	32
Threshold	ND-CTR	48.5 ± 1.0	44.4 ± 1.1	37.9 ± 1.2	38.7 ± 1.4	37.3 ± 1.5	41.1 ± 1.7	43.9 ± 1.9
	ND-1D	77.8 ± 1.7	78.3 ± 1.2	78.3 ± 1.2	79.4 ± 0.6	80.0 ± 0.0	78.9 ± 0.7	78.3 ± 0.8
	ND-10D	75.7 ± 1.4	76.1 ± 1.3	75.4 ± 1.9	78.2 ± 0.8	77.5 ± 1.0	76.8 ± 1.4	76.8 ± 1.0
	ND-30D	80.0 ± 0.0	80.0 ± 0.0	80.0 ± 0.0	80.0 ± 0.0	80.0 ± 0.0	79.3 ± 0.7	79.3 ± 0.7
	ED-CTR	47.4 ± 1.0	42.6 ± 1.1	37.1 ± 1.3	37.8 ± 1.3	37.4 ± 1.5	39.6 ± 1.3	42.4 ± 1.4
	ED-1D	72.9 ± 2.9	70.7 ± 3.4	69.3 ± 4.0	74.3 ± 1.7	73.6 ± 4.0	72.9 ± 3.9	73.6 ± 2.1
	ED-10D	62.0 ± 1.9	59.0 ± 2.1	54.0 ± 2.3	60.5 ± 2.3	60.0 ± 2.2	61.0 ± 1.9	60.5 ± 1.6
	ED-30D	61.7 ± 2.1	62.5 ± 1.1	55.0 ± 5.3	59.2 ± 3.0	61.7 ± 1.1	65.0 ± 1.8	64.2 ± 2.7
**Anova: *F*_(7,40)_ =**		46.9 (***)	62.8 (***)	29.2 (***)	86.9 (***)	66.8 (***)	57.5 (***)	84.3 (***)
		**0.5**	**1**	**2**	**4**	**8**	**16**	**32**
Threshold Shift	ND-1D	30.0 ± 1.7	36.7 ± 1.9	40.6 ± 1.9	44.4 ± 2.4	46.0 ± 1.8	42.8 ± 1.5	40.6 ± 1.3
	ND-10D	22.5 ± 2.7	26.0 ± 4.0	32.5 ± 4.4	35.0 ± 3.2	37.5 ± 3.0	32.0 ± 2.7	30.8 ± 3.9
	ND-30D	25.5 ± 2.5	28.9 ± 3.6	35.5 ± 4.9	38.0 ± 2.8	40.0 ± 2.5	36.0 ± 3.2	31.8 ± 3.3
	ED-1D	21.4 ± 3.0	25.0 ± 3.8	30.9 ± 5.4	33.6 ± 3.6	30.7 ± 3.8	30.0 ± 4.5	29.3 ± 3.0
	ED-10D	15.5 ± 1.7	19.0 ± 2.4	20.0 ± 3.3	26.0 ± 1.9	25.5 ± 2.6	23.5 ± 2.8	23.0 ± 2.5
	ED-30D	17.0 ± 2.5	20.0 ± 2.4	19.0 ± 2.2	25.5 ± 2.7	26.5 ± 3.0	24.0 ± 2.3	22.0 ± 2.1
**ANOVA: *F*_(5,30 )_=**		23.5 (***)	5.6 (***)	5.2 (***)	8.6 (***)	6.8 (***)	5.8 (***)	9.5 (***)

*** *p* < 0.001.

**Table 4:** Mean + SE and ANOVA of the interaction between diet, noise over exposure and percentage of OHC survival in the cochlea, relative to the control condition.

Groups	Apical			Middle			Basal	
**ND-CTR**	100.00 ± 0.00			100.00 ± 0.00			100.00 ± 0.00	
**ND-1D**	92.04 ± 1.21			63.45 ± 1.68			73.12 ± 2.35	
**ND-10D**	90.16 ± 2.12			53.79 ± 2.93			67.43 ± 0.97	
**ND-30D**	81.73 ± 1.22			41.61 ± 2.20			61.28 ± 2.46	
**ED-CTR**	100.00 ± 0.00			100.00 ± 0.00			100.00 ± 0.00	
**ED-1D**	97.20 ± 1.23			75.12 ± 1.16			86.89 ± 2.26	
**ED-10D**	96.73 ± 1.53			67.74 ± 2.46			80.80 ± 0.99	
**ED-30D**	93.46 ± 1.49			64.29 ± 1.29			78.22 ± 1.11	
**Anova:**	***F*_(7,48)_** = 13.8 (***)			***F*_(7,48)_** = 97.9 (***)			***F*_(7,48)_** = 41.9 (***)	
**Significance levels**								
***Apical***	**ND-CTR**	**ED-CTR**	**ND-1D**	**ED-1D**	**ND-10D**	**ED-10D**	**ND-30D**	**ED-30D**
**ND-CTR**		1.000	0.311	0.989	0.025	0.971	0.000	0.406
**ED-CTR**	1.000		0.269	0.982	0.020	0.957	0.000	0.357
**ND-1D**	0.311	0.269		0.852	0.971	0.915	0.000	1.000
**ED-1D**	0.989	0.982	0.852		0.230	1.000	0.000	0.915
**ND-10D**	0.025	0.020	0.971	0.230		0.311	0.094	0.938
**ED-10D**	0.971	0.957	0.915	1.000	0.311		0.000	0.957
**ND-30D**	0.000	0.000	0.000	0.000	0.094	0.000		0.000
**ED-30D**	0.406	0.357	1.000	0.915	0.938	0.957	0.000	
***Middle***	**ND-CTR**	**ED-CTR**	**ND-1D**	**ED-1D**	**ND-10D**	**ED-10D**	**ND-30D**	**ED-30D**
**ND-CTR**		1.000	0.000	0.000	0.000	0.000	0.000	0.000
**ED-CTR**	1.000		0.000	0.000	0.000	0.000	0.000	0.000
**ND-1D**	0.000	0.000		0.049	0.008	0.994	0.000	1.000
**ED-1D**	0.000	0.000	0.049		0.000	0.415	0.000	0.046
**ND-10D**	0.000	0.000	0.008	0.000		0.003	0.014	0.066
**ED-10D**	0.000	0.000	0.994	0.415	0.003		0.000	0.976
**ND-30D**	0.000	0.000	0.000	0.000	0.014	0.000		0.000
**ED-30D**	0.000	0.000	1.000	0.046	0.066	0.976	0.000	
***Basal***	**ND-CTR**	**ED-CTR**	**ND-1D**	**ED-1D**	**ND-10D**	**ED-10D**	**ND-30D**	**ED-30D**
**ND-CTR**		0.992	0.000	0.000	0.000	0.000	0.000	0.000
**ED-CTR**	0.992		0.000	0.000	0.000	0.000	0.000	0.000
**ND-1D**	0.000	0.000		0.034	0.551	0.845	0.000	0.998
**ED-1D**	0.000	0.000	0.034		0.000	0.644	0.000	0.194
**ND-10D**	0.000	0.000	0.551	0.000		0.023	0.598	0.168
**ED-10D**	0.000	0.000	0.845	0.644	0.023		0.000	0.995
**ND-30D**	0.000	0.000	0.000	0.000	0.598	0.000		0.000
**ED-30D**	0.000	0.000	0.998	0.194	0.168	0.995	0.000	

*** *p* < 0.001.

**Table 5:** Mean + SE and ANOVA of the interaction between diet, noise over exposure, and expression of antioxidant enzymes genes in the cochlea.

Groups	Apical			Middle			Basal	
**ND-CTR**	100.00 ± 0.00			100.00 ± 0.00			100.00 ± 0.00	
**ND-1D**	92.04 ± 1.21			63.45 ± 1.68			73.12 ± 2.35	
**ND-10D**	90.16 ± 2.12			53.79 ± 2.93			67.43 ± 0.97	
**ND-30D**	81.73 ± 1.22			41.61 ± 2.20			61.28 ± 2.46	
**ED-CTR**	100.00 ± 0.00			100.00 ± 0.00			100.00 ± 0.00	
**ED-1D**	97.20 ± 1.23			75.12 ± 1.16			86.89 ± 2.26	
**ED-10D**	96.73 ± 1.53			67.74 ± 2.46			80.80 ± 0.99	
**ED-30D**	93.46 ± 1.49			64.29 ± 1.29			78.22 ± 1.11	
**Anova:**	***F*_(7,48)_** = 13.8 (***)			***F*_(7,48)_** = 97.9 (***)			***F*_(7,48)_** = 41.9 (***)	
**Significance levels**								
***Apical***	**ND-CTR**	**ED-CTR**	**ND-1D**	**ED-1D**	**ND-10D**	**ED-10D**	**ND-30D**	**ED-30D**
**ND-CTR**		1.000	0.311	0.989	0.025	0.971	0.000	0.406
**ED-CTR**	1.000		0.269	0.982	0.020	0.957	0.000	0.357
**ND-1D**	0.311	0.269		0.852	0.971	0.915	0.000	1.000
**ED-1D**	0.989	0.982	0.852		0.230	1.000	0.000	0.915
**ND-10D**	0.025	0.020	0.971	0.230		0.311	0.094	0.938
**ED-10D**	0.971	0.957	0.915	1.000	0.311		0.000	0.957
**ND-30D**	0.000	0.000	0.000	0.000	0.094	0.000		0.000
**ED-30D**	0.406	0.357	1.000	0.915	0.938	0.957	0.000	
***Middle***	**ND-CTR**	**ED-CTR**	**ND-1D**	**ED-1D**	**ND-10D**	**ED-10D**	**ND-30D**	**ED-30D**
**ND-CTR**		1.000	0.000	0.000	0.000	0.000	0.000	0.000
**ED-CTR**	1.000		0.000	0.000	0.000	0.000	0.000	0.000
**ND-1D**	0.000	0.000		0.049	0.008	0.994	0.000	1.000
**ED-1D**	0.000	0.000	0.049		0.000	0.415	0.000	0.046
**ND-10D**	0.000	0.000	0.008	0.000		0.003	0.014	0.066
**ED-10D**	0.000	0.000	0.994	0.415	0.003		0.000	0.976
**ND-30D**	0.000	0.000	0.000	0.000	0.014	0.000		0.000
**ED-30D**	0.000	0.000	1.000	0.046	0.066	0.976	0.000	
***Basal***	**ND-CTR**	**ED-CTR**	**ND-1D**	**ED-1D**	**ND-10D**	**ED-10D**	**ND-30D**	**ED-30D**
**ND-CTR**		0.992	0.000	0.000	0.000	0.000	0.000	0.000
**ED-CTR**	0.992		0.000	0.000	0.000	0.000	0.000	0.000
**ND-1D**	0.000	0.000		0.034	0.551	0.845	0.000	0.998
**ED-1D**	0.000	0.000	0.034		0.000	0.644	0.000	0.194
**ND-10D**	0.000	0.000	0.551	0.000		0.023	0.598	0.168
**ED-10D**	0.000	0.000	0.845	0.644	0.023		0.000	0.995
**ND-30D**	0.000	0.000	0.000	0.000	0.598	0.000		0.000
**ED-30D**	0.000	0.000	0.998	0.194	0.168	0.995	0.000	

*** *p* < 0.001.

**Table 6:** Mean + SE and ANOVA of the interaction between diet, noise over exposure, and expression of apoptosis genes in the cochlea.

Groups	*Bax*			*Bcl-2*			*Casp3*	
**ND-CTR**	0.95 ± 0.04			1.02 ± 0.03			0.95 ± 0.03	
**ND-1D**	0.99 ± 0.04			1.00 ± 0.03			1.30 ± 0.05	
**ND-10D**	1.63 ± 0.09			1.55 ± 0.07			1.71 ± 0.07	
**ND-30D**	1.14 ± 0.04			1.13 ± 0.04			1.24 ± 0.07	
**ED-CTR**	1.02 ± 0.03			1.01 ± 0.04			1.00 ± 0.01	
**ED-1D**	0.89 ± 0.02			0.92 ± 0.04			1.22 ± 0.04	
**ED-10D**	1.42 ± 0.05			1.90 ± 0.08			1.32 ± 0.04	
**ED-30D**	0.86 ± 0.02			1.05 ± 0.04			1.06 ± 0.02	
**Anova:**	*F*_(7,274)_ = 35.7 (***)			*F*_(7,254)_ = 45.8 (***)			*F*_(7,266)_ = 35.8 (***)	
**Significance levels**								
***Bax***	**ND-CTR**	**ED-CTR**	**ND-1D**	**ED-1D**	**ND-10D**	**ED-10D**	**ND-30D**	**ED-30D**
**ND-CTR**		0.922	0.999	0.984	0.000	0.000	0.161	0.886
**ED-CTR**	0.922		1.000	0.346	0.000	0.000	0.793	0.162
**ND-1D**	0.999	1.000		0.896	0.000	0.000	0.675	0.722
**ED-1D**	0.984	0.346	0.896		0.000	0.000	0.014	1.000
**ND-10D**	0.000	0.000	0.000	0.000		0.249	0.000	0.000
**ED-10D**	0.000	0.000	0.000	0.000	0.249		0.006	0.000
**ND-30D**	0.161	0.793	0.675	0.014	0.000	0.006		0.005
**ED-30D**	0.886	0.162	0.722	1.000	0.000	0.000	0.005	
***Bcl-2***	**ND-CTR**	**ED-CTR**	**ND-1D**	**ED-1D**	**ND-10D**	**ED-10D**	**ND-30D**	**ED-30D**
**ND-CTR**		1.000	1.000	0.954	0.000	0.000	0.946	1.000
**ED-CTR**	1.000		1.000	0.952	0.000	0.000	0.886	1.000
**ND-1D**	1.000	1.000		0.994	0.000	0.000	0.917	1.000
**ED-1D**	0.954	0.952	0.994		0.000	0.000	0.373	0.823
**ND-10D**	0.000	0.000	0.000	0.000		0.009	0.003	0.000
**ED-10D**	0.000	0.000	0.000	0.000	0.009		0.000	0.000
**ND-30D**	0.946	0.886	0.917	0.373	0.003	0.000		0.988
**ED-30D**	1.000	1.000	1.000	0.823	0.000	0.000	0.988	
***Casp3***	**ND-CTR**	**ED-CTR**	**ND-1D**	**ED-1D**	**ND-10D**	**ED-10D**	**ND-30D**	**ED-30D**
**ND-CTR**		0.981	0.000	0.000	0.000	0.000	0.000	0.486
**ED-CTR**	0.981		0.000	0.004	0.000	0.000	0.003	0.951
**ND-1D**	0.000	0.000		0.955	0.000	1.000	0.997	0.010
**ED-1D**	0.000	0.004	0.955		0.000	0.745	1.000	0.222
**ND-10D**	0.000	0.000	0.000	0.000		0.000	0.000	0.000
**ED-10D**	0.000	0.000	1.000	0.745	0.000		0.949	0.002
**ND-30D**	0.000	0.003	0.997	1.000	0.000	0.949		0.149
**ED-30D**	0.486	0.951	0.010	0.222	0.000	0.002	0.149	

*** *p* < 0.001.
